# Nucleobindin-2 consists of two structural components: The Zn^2+^-sensitive N-terminal half, consisting of nesfatin-1 and -2, and the Ca^2+^-sensitive C-terminal half, consisting of nesfatin-3

**DOI:** 10.1016/j.csbj.2021.07.036

**Published:** 2021-07-30

**Authors:** Dominika Bystranowska, Anna Skorupska, Katarzyna Sołtys, Michał Padjasek, Artur Krężel, Andrzej Żak, Magdalena Kaus-Drobek, Michał Taube, Maciej Kozak, Andrzej Ożyhar

**Affiliations:** a Department of Biochemistry, Molecular Biology and Biotechnology, Faculty of Chemistry, Wrocław University of Science and Technology, Wybrzeże Wyspiańskiego 27, 50-370 Wrocław, Poland; b Department of Chemical Biology, Faculty of Biotechnology, University of Wrocław, Joliot-Curie 14a, 50-383 Wrocław, Poland; c Faculty of Mechanical Engineering, Wrocław University of Science and Technology, Wybrzeże Wyspiańskiego 27, 50-370 Wrocław, Poland; d Institute of Biochemistry and Biophysics, Polish Academy of Sciences, 02-106 Warsaw, Poland; e Department of Macromolecular Physics, Faculty of Physics, Adam Mickiewicz University, Umultowska 85, 61-614 Poznań, Poland; f National Synchrotron Radiation Centre SOLARIS, Jagiellonian University, Czerwone Maki 98, 30-392 Kraków, Poland

**Keywords:** Intrinsically disordered proteins, Zinc ion binding proteins, Oligomers, Analytical ultracentrifugation, Transmission electron microscopy, Isothermal titration calorimetry

## Abstract

Nucleobindin-2 (Nucb2) is a protein that has been suggested to play roles in a variety of biological processes. Nucb2 contains two Ca^2+^/Mg^2+^-binding EF-hand domains separated by an acidic amino acid residue-rich region and a leucine zipper. All of these domains are located within the C-terminal half of the protein. At the N-terminal half, Nucb2 also possesses a putative Zn^2+^-binding motif. In our recent studies, we observed that Nucb2 underwent Ca^2+^-dependent compaction and formed a mosaic-like structure consisting of intertwined disordered and ordered regions at its C-terminal half. The aim of this study was to investigate the impact of two other potential ligands: Mg^2+^, which possesses chemical properties similar to those of Ca^2+^, and Zn^2+^, for which a putative binding motif was identified. In this study, we demonstrated that the binding of Mg^2+^ led to oligomerization state changes with no significant secondary or tertiary structural alterations of Nucb2. In contrast, Zn^2+^ binding had a more pronounced effect on the structure of Nucb2, leading to the local destabilization of its N-terminal half while also inducing changes within its C-terminal half. These structural rearrangements resulted in the oligomerization and/or aggregation of Nucb2 molecules. Taken together, the results of our previous and current research help to elucidate the structure of the Nucb2, which can be divided into two parts: the Zn^2+^-sensitive N-terminal half (consisting of nesfatin-1 and -2) and the Ca^2+^-sensitive C-terminal half (consisting of nesfatin-3). These results may also help to open a new discussion regarding the diverse roles that metal cations play in regulating the structure of Nucb2 and the various physiological functions of this protein.

## Graphical abstract

**Figure:**
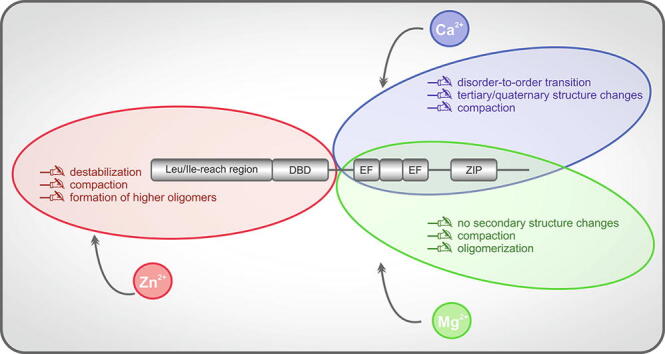

## 1 Introduction

Metal ions have a significant impact on a variety of biological processes. In fact, these ions are essential for the functioning of approximately one-third of proteins. Metal-protein interactions are involved in the conformational alternation of the structure of proteins and the catalysis of biological processes [1]. Ca^2+^, Mg^2+^ and Zn^2+^, which are divalent metal cations, are among the most important metal ions due to their abundance in the body [2], [3], [4]. Ca^2+^, a ubiquitous secondary messenger, regulates relevant processes from cell signalling to apoptosis [5]. The most common Ca^2+^-binding motif in proteins is the EF-hand domain [6], [7]. Another essential metal ion, Mg^2+^, possesses chemical properties similar to those of Ca^2+^ and is also able to bind to the EF-hand domain [6]. However, the cytosolic concentration of Mg^2+^ is approximately three orders of magnitude higher than that of Ca^2+^ [8]. Due to the different ionic radii of Mg^2+^ (0.86 Å) and Ca^2+^ (1.14 Å), proteins with EF-hand domains can differentiate Ca^2+^ from Mg^2+^ [9].

Zn^2+^, one of the most abundant d-block metal ions, is essential as a cofactor for the enzymes and structural or regulatory elements of proteins [10]. Bioinformatic analysis revealed that approximately 10% of proteins in the human genome are involved in Zn^2+^ binding [11]. Unlike Ca^2+^ and Mg^2+^, Zn^2+^ has a stronger affinity for proteins [12]. Zn^2+^ ions can bind between four to six protein ligands, such as the nitrogen atoms of His, sulfur atoms of Cys and oxygen atoms of Asp and Glu residues, and form complexes with tetrahedral to octahedral geometry [10], [13]. The most classical structural Zn^2+^-binding site is formed by the Cys2His2 coordination environment in the ββα zinc finger domain [14]. However, other structural Zn^2+^-binding sites, such as Cys4, Cys3His, AspHis3, and His2Asp2, are also common [15], [16]. Zn^2+^ is also implicated in cellular regulation [3], [17]. The total Zn^2+^ concentration in eukaryotic cells is several hundred micromolar. However, the free Zn^2+^ concentration is considerably lower, being in the picomolar to sub-nanomolar range [12], [18]. The concentration of Zn^2+^ must be tightly regulated, with both an abundance and deficiency of Zn^2+^ being adverse for cells [19], [20]. Multiple proteins (e.g., the metallothionein family) are involved in Zn^2+^ homeostasis to keep the free Zn^2+^ concentration at the proper range and to provide this element for multiple Zn^2+^-binding proteins [21]. Compartmentation of Zn^2+^ in different cellular organelles by Zn^2+^ transporters is also characteristic of the control of Zn^2+^ levels in eukaryotic cells [12], [22]. The vesicles of neurons and pancreatic β cells are examples of organelles that store large amounts of Zn^2+^ [23], [24]. The concentration of free Zn^2+^ is increased locally by the vesicular release of Zn^2+^ [25]. This Zn^2+^ increase participates in protein function regulation and controls various cellular processes implicated in signal transduction [26]. Importantly, dysregulated changes that lead to Zn^2+^ dyshomeostasis are related to several neurodegenerative diseases [27] and mental disorders, such as depression [28]. The binding of Zn^2+^ affects the aggregation kinetics and morphology of a high percentage of amyloid proteins, such as amyloid-β [29], [30], Tau [31], islet amyloid polypeptide [32], [33], β2-microglobulin [34] and superoxide dismutase 1 [35]. A high Zn^2+^ concentration could also lead to protein precipitation and modulate the toxicity of protein aggregates [36].

Nucleobindin-2 (Nucb2) is a multidomain protein consisting of six domains: the Ile/Leu-rich region, the DNA-binding domain (DBD), two EF-hand domains, the acidic amino acid rich region, and the leucine zipper motif [37]. Nucb2 can be proteolytically cleaved into three peptides: nesfatin-1, nesfatin-2, and nesfatin-3 [38]. Only the function of nesfatin-1 has been determined [38]. Oh-I [38] showed that nesfatin-1 is involved in food intake inhibition [38]. Nucb2 also participates in other biological processes, e.g., adipogenesis [39], biomineralization [40] and carcinogenesis [41], [42]. Additionally, Nucb2 is ubiquitously expressed in the central nervous system [38] and peripheral tissues, such as the digestive tract [43], reproductive system [44], pancreas [45], [46], and heart [47]. The multifunctionality of Nucb2 may result from its structure. Nucb2 possesses two EF-hand domains [37], which are able to bind Ca^2+^ and Mg^2+^ ions [6]. Additionally, Nucb2 contains a putative Zn^2+^-binding motif at its N-terminal half, similar to its paralogue Nucb1 [48]. Our previous research showed that Nucb2s from *Gallus gallus* (ggNucb2) and *Homo sapiens* (hsNucb2) belong to the family of intrinsically disordered proteins (IDPs) [49]. We also revealed that Ca^2+^ binding affects the overall structure of Nucb2 molecules, which leads to their compaction with the simultaneous formation of a mosaic-like structure consisting of intertwined disordered and ordered regions of the initially disordered C-terminal half of apo-Nucb2 [49]. However, the impact of Mg^2+^ and Zn^2+^ on the structure of Nucb2s has not been examined to date. Therefore, the aim of this study was to characterize the structural properties of both Nucb2 homologues in the presence of Zn^2+^ and Mg^2+^ using a range of biophysical experimental approaches. In this study, we demonstrate the presence of two Zn^2+^-binding sites and a single Mg^2+^-binding site within Nucb2 molecules. Moreover, the results of this study indicate that Mg^2+^ or Zn^2+^ binding leads to conformational changes in the structures of both proteins. Induced by Zn^2+^ binding, destabilization of the structure of Nucb2s within the N-terminal half leads to the self-assembly of the proteins’ molecules and the formation of higher oligomers. On the other hand, Mg^2+^ binding does not induce significant changes in the secondary/tertiary structure of Nucb2s. However, Mg^2+^ impacts the oligomerization state of Nucb2s, leading to the appearance of ggNucb2 dimeric species and the slightly compacted molecules of hsNucb2. The results presented in this report provide comprehensive insight into the structure of Nucb2 molecules, which appear to be divided into two parts: the Zn^2+^-sensitive N-terminal half, consisting of nesfatin-1 and -2, and the Ca^2+^-sensitive C-terminal half, which consists of nesfatin-3.

## 2 Results

### 2.1 Secondary structure modulation of Nucb2s revealed by CD

**Fig. 1:**
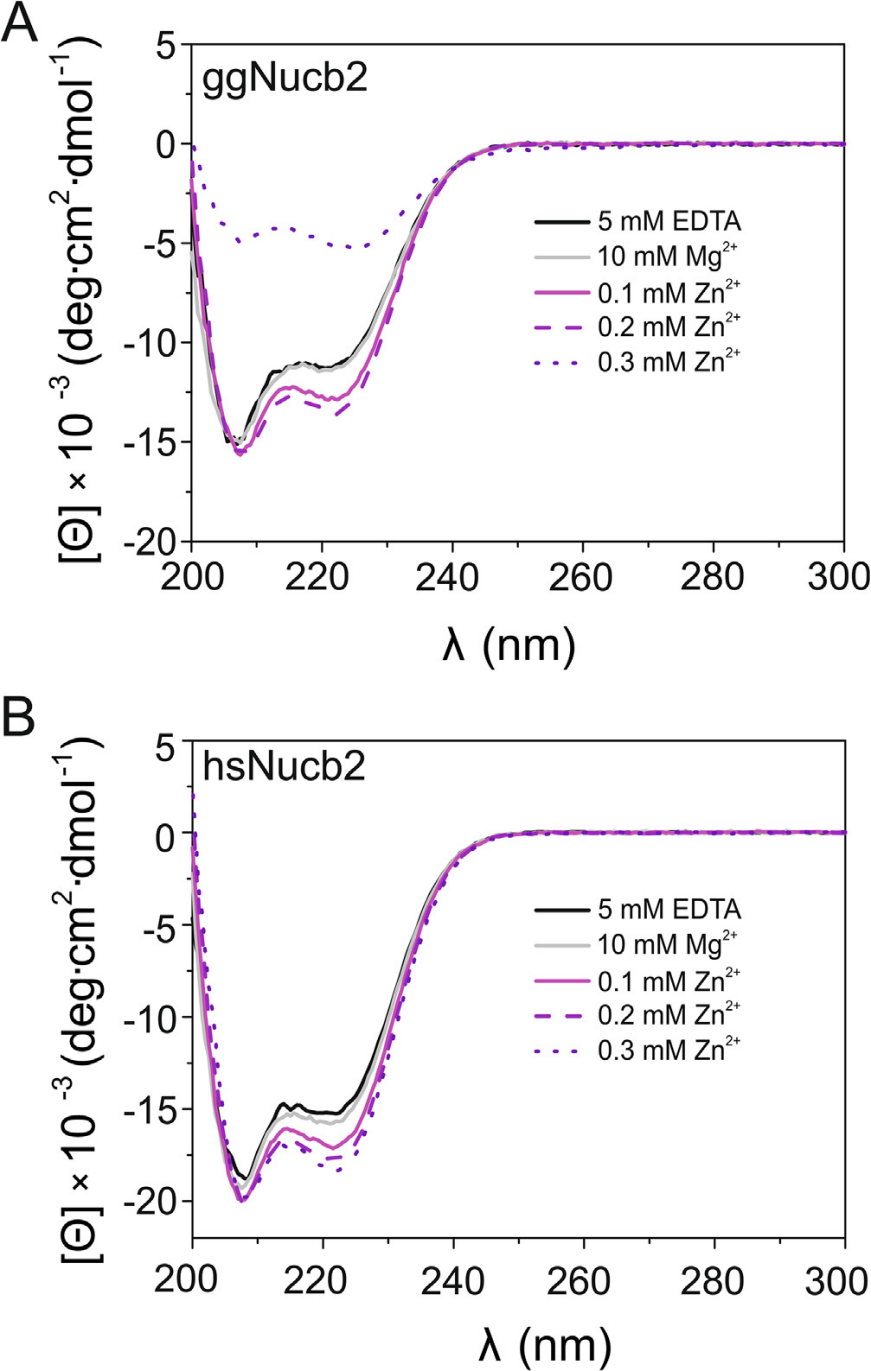
Effect of Zn^2+^ and Mg^2+^ on the secondary structure of Nucb2s. (A) Far-UV CD spectra of ggNucb2 (10 µM) in buffer C in the presence of 5 mM EDTA (solid black line), in the presence of 10 mM MgCl_2_ (solid grey line), in the presence of 100 µM ZnCl_2_ (solid magenta line), in the presence of 200 µM ZnCl_2_ (dashed purple line), and in the presence of 300 µM ZnCl_2_ (dotted violet line). (B) Far-UV CD spectra of hsNucb2 (10 µM) in buffer C in the presence of 5 mM EDTA (solid black line), in the presence of 10 mM MgCl_2_ (solid grey line), in the presence of 100 µM ZnCl_2_ (solid magenta line), in the presence of 200 µM ZnCl_2_ (dashed purple line), and in the presence of 300 µM ZnCl_2_ (dotted violet line). The data shown are representative of three independent experiments. (For interpretation of the references to colour in this figure legend, the reader is referred to the web version of this article.)

Previously [49], we showed that in the presence of Ca^2+^, ggNucb2 and hsNucb2 undergo a significant compaction of their structure, including an increase in the content of the α-helical structure, while exhibiting a simultaneous decrease in the fraction of random coil regions. In this study, we examined other divalent metal ions – Zn^2+^ and Mg^2+^ – as well as their effect on Nucb2 secondary structures. In the first step, we performed far-UV CD spectra measurements of Nucb2s in the absence and presence of 100–300 µM Zn^2+^ and 10 mM Mg^2+^. Such a concentration of Mg^2+^ was chosen due to the mM range of the physiological concentration of Mg^2+^ [4]. The estimates of the total intracellular Mg^2+^ concentration are 15 mM, whereas the ionized intracellular Mg^2+^ concentration ranges between 0.5 and 1.0 mM [50]. We decided to use a µM Zn^2+^ concentration because Zn^2+^ within the µM range can accumulate in a variety of cells and tissues, e.g., neurons [51] and pancreatic β-cells [52], under physiological conditions, despite the low free Zn^2+^ concentration (pM to nM range). Additionally, pathological states are also related to the accumulation of Zn^2+^ at µM concentrations [53], [54]. As shown in Fig. 1A and 1B, the CD spectra of both apo-Nucb2s show typical negative maxima for the α-helical structure at 208 nm and 222 nm [55]. These results were supported by deconvolution of CD data using CDNN software [56], which shows that Nucb2s consist primarily of the α-helical structure (ggNucb2 – 45.10 ± 0.01%, hsNucb2 – 55.60 ± 0.27%, see Table 1). The analysis also indicated the presence of a significant number of random coil regions (ggNucb2 – 25.60 ± 1.5%, hsNucb2 – 20.3 ± 0.2%). The presence of 100 µM and 200 µM Zn^2+^ was observed to slightly modulate the secondary structure composition of Nucb2s, leading to an increase in the α-helical structure content and a decrease in the random coil content (Table 1 and Fig. 1A and B). Notably, incubation of ggNucb2 with 300 µM Zn^2+^ caused the CD signal to decay (Fig. 1A). Conversely, the addition of 300 µM Zn^2+^ had a similar effect on the structure of hsNucb2 as the addition of 200 µM Zn^2+^ (Fig. 1B). At the protein concentration that was employed and with increasing concentrations of Zn^2+^ (≥300 µM), the measurements performed for ggNucb2 were observed to be more problematic due to the precipitation of the protein compared with the measurements performed for hsNucb2. On the other hand, Mg^2+^ had no impact on the secondary structure of ggNucb2. The spectra of ggNucb2 in the absence and presence of 10 mM Mg^2+^ were determined to be superimposable (see Fig. 1A). Mg^2+^ affected, however, the secondary structure of hsNucb2. The deconvoluted CD spectra showed that the effect of Mg^2+^ on the secondary structure of hsNucb2 was similar to the effect of 100 µM Zn^2+^, as addition of both cations induced a slight increase in the α-helical structure content of hsNucb2 (Fig. 1B and Table 1).

**Table 1:** Secondary structure composition of Nucb2s in the absence and presence of Zn^2+^ and Mg^2+^. The table shows the content (%) of each secondary structural element in the Nucb2s estimated by CDNN [56]. The results collected for ggNucb2 in the presence of 300 µM Zn^2+^ (*) do not sum to 100% and should be treated with caution due to partial protein precipitation. The results are the average of three independent experiments.

Agent	α-helix	Antiparallel β-sheet	Parallel β-sheet	β-turn	Random coil
	[%]				
ggNucb2					
5 mM EDTA	45.1 ± 0.0	5.7 ± 0.0	6.3 ± 0.3	15.1 ± 0.0	25.6 ± 1.5
100 µM ZnCl_2_	49.8 ± 0.5	5.2 ± 0.1	5.5 ± 0.1	14.4 ± 0.1	23.2 ± 0.3
200 µM ZnCl_2_	51.7 ± 0.8	4.9 ± 0.1	5.2 ± 0.1	14.1 ± 0.1	22.3 ± 0.4
300 µM ZnCl_2_*	18.7 ± 0.0	12.6 ± 0.0	15.4 ± 0.1	19.7 ± 0.0	46.6 ± 0.0
10 mM MgCl_2_	43.5 ± 2.0	6.0 ± 0.3	6.6 ± 0.4	15.3 ± 0.3	26.5 ± 1.2

hsNucb2					
5 mM EDTA	55.6 ± 0.3	4.4 ± 0.0	4.6 ± 0.1	13.7 ± 0.0	20.3 ± 0.2
100 µM ZnCl_2_	60.5 ± 0.2	3.9 ± 0.6	3.9 ± 0.6	13.0 ± 0.6	18.0 ± 0.6
200 µM ZnCl_2_	62.4 ± 0.5	3.7 ± 0.6	3.7 ± 0.1	12.8 ± 0.6	17.2 ± 0.2
300 µM ZnCl_2_	62.8 ± 0.3	3.6 ± 0.0	3.7 ± 0.1	12.7 ± 0.1	17.2 ± 0.2
10 mM MgCl_2_	59.4 ± 0.6	4.0 ± 0.0	4.1 ± 0.0	13.2 ± 0.0	18.5 ± 0.0

### 2.2 Tertiary structure change of holo-Nucb2s revealed by fluorescence spectroscopy

**Fig. 2:**
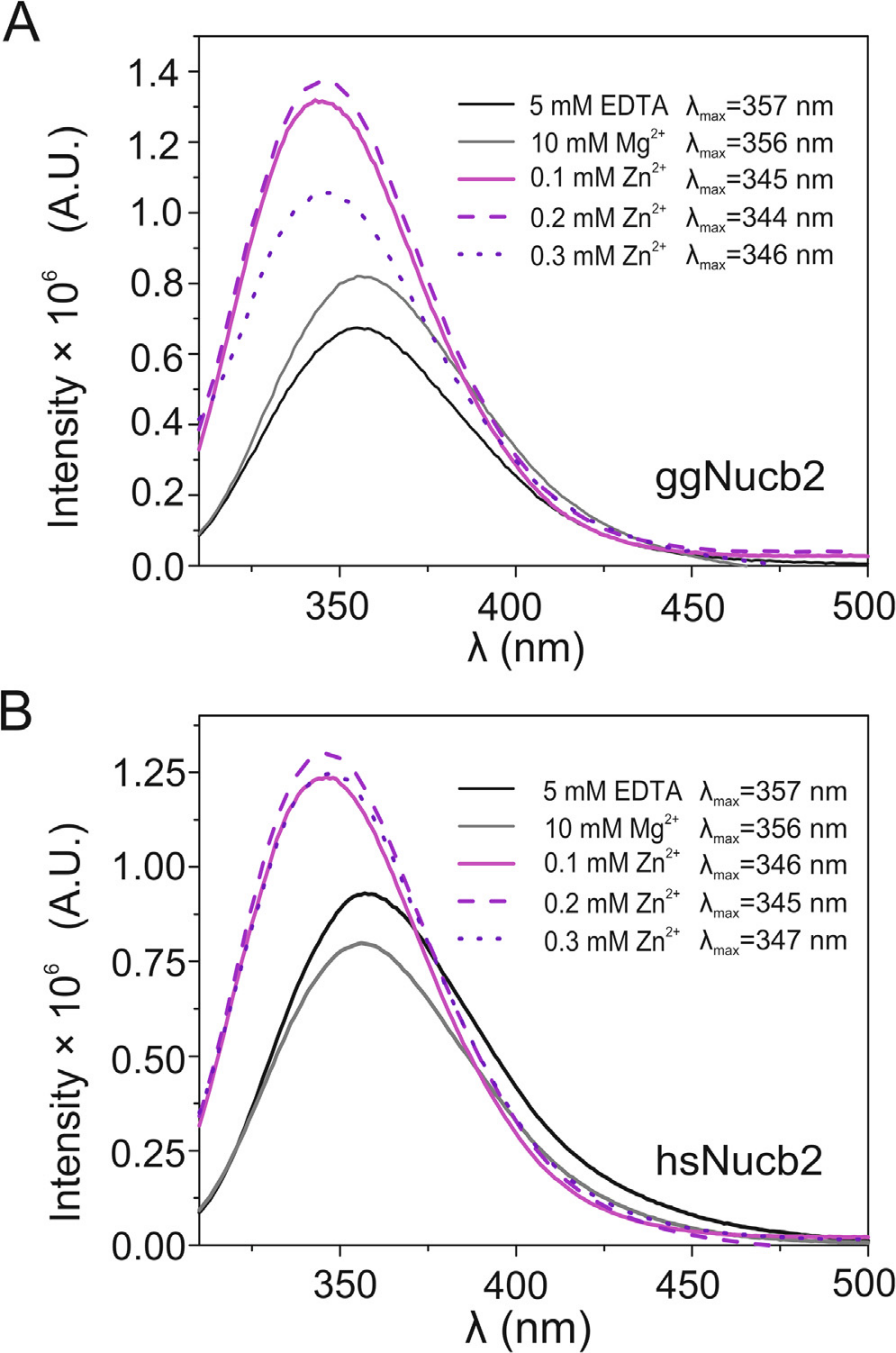
Intrinsic fluorescence spectra of Nucb2s in the presence of 5 mM EDTA and 10 mM Mg^2+^ and in the presence of an increasing concentration of Zn^2+^ (100–300 µM). The fluorescence spectra of (A) ggNucb2 and (B) hsNucb2. The protein concentration was 2 µM. The measurements were performed at 20 °C.

CD spectra analysis revealed that Zn^2+^ slightly modified the secondary structure of Nucb2s, thereby leading to an increase in the α-helical content. To investigate possible changes in the tertiary structure, we utilized the intrinsic fluorescence of Trp residues. The emission spectrum of Trp residues depends on the polarity of their environment [57]. The two Trp residues present in the structure of both Nucb2 molecules were determined to be fully exposed to the solvent in the absence of metal cations, since the emission maxima were observed at 355 nm and 357 nm for ggNucb2 and hsNucb2, respectively (Fig. 2A, Fig. 2B). This property is characteristic of Trp residues located within the inherently disordered regions (IDRs) of proteins [57]. The addition of 100–300 µM ZnCl_2_, however, led to a visible blueshift of the fluorescence maxima of 10 nm (to 345 nm and 347 nm for ggNucb2 and hsNucb2, respectively), suggesting that the polarity of the microenvironment of the Trp residues changed to become more hydrophobic after Zn^2+^ binding (see Fig. 2A and 2B). Additionally, the fluorescence intensity of the Trp residues of ggNucb2 in the presence of 300 µM Zn^2+^ decreased, which might also be attributable to the precipitation of protein. Notably, the presence of Mg^2+^ has no impact on the position of the Trp emission maxima of Nucb2s. These data indicate that the presence of Zn^2+^ impacts the tertiary structure of both Nucb2s, which can be explained by the notable structural rearrangement of the corresponding regions, which probably leads to the compaction of the protein molecules.

### 2.3 Nucb2 quaternary structure changes induced by divalent metal cations observed by SV-AUC

**Fig. 3:**
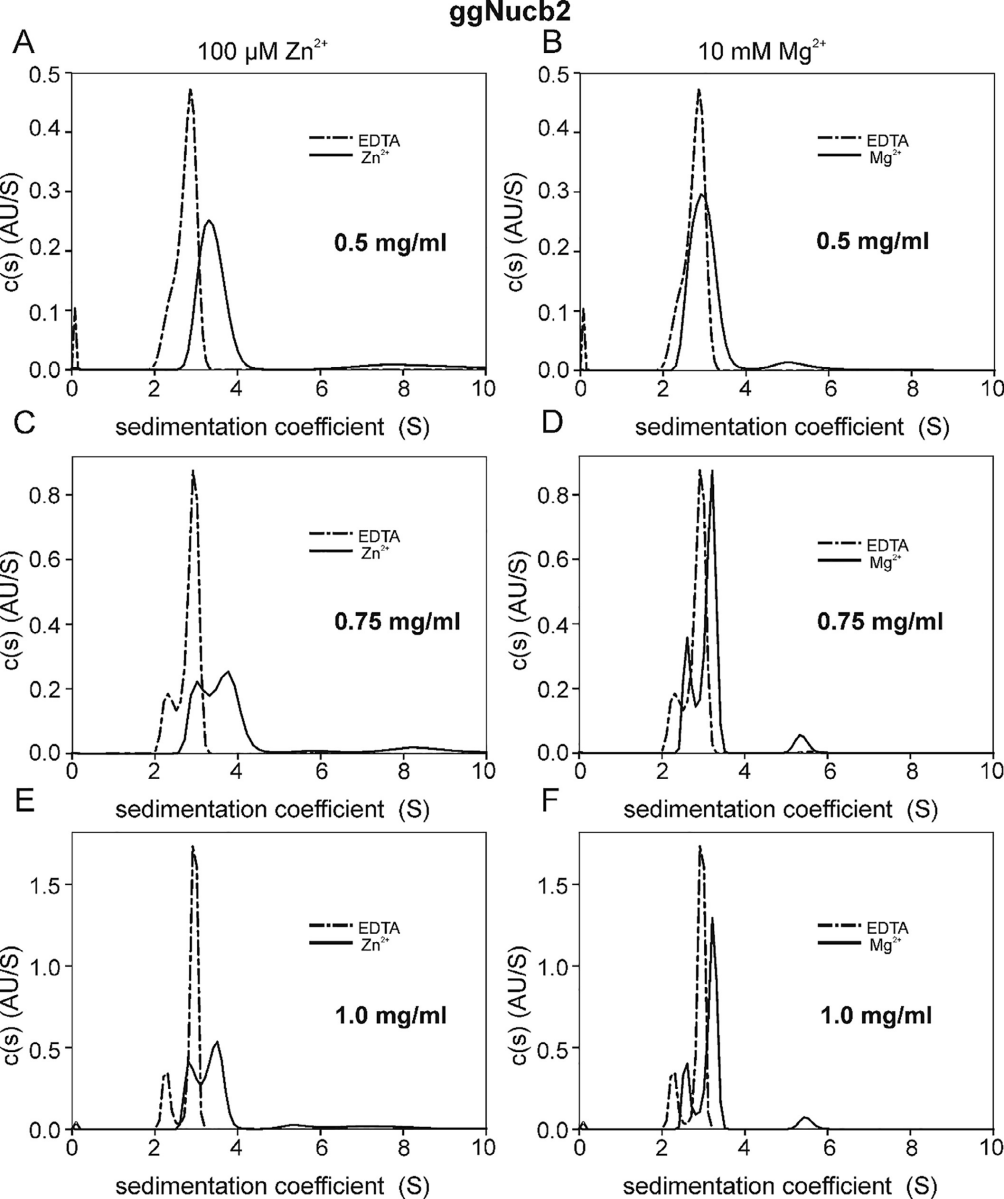
Size distribution characterization of ggNucb2 in the absence and presence of Zn^2+^ and Mg^2+^. The SV-AUC measurements were performed for three concentrations of ggNucb2: 0.5 (A, B), 0.75 (C, D) and 1.0 mg/ml (E, F) in the presence of 5 mM EDTA, 10 mM Mg^2+^ and 100 µM Zn^2+^, at 50 000 rpm and at 20 °C. Dash-dotted line – EDTA, solid line – appropriate metal ion.

We recently reported [49] that Nucb2s exist in solution as extended molecules with the propensity for dimerization and simultaneous compaction induced by Ca^2+^ binding. These flexible characteristics of the Nucb2 structure were thought to play important roles in the variety of Ca^2+^-dependent interactions with different protein partners [58], [59]. In this study, we investigated how the conformation of Nucb2 molecules changes upon binding Zn^2+^ and Mg^2+^ and whether these divalent metal cations influence the quaternary structure of Nucb2. Using SV-AUC, we observed that the sedimentation profiles of Nucb2s fit a continuous c(s) distribution model and that major peaks of s_(20,w)_ = 3.08 S and s_(20,w)_ = 3.39 S (Table 2) were observed at the highest protein concentration and in the absence of metal cations, respectively. The major population of molecules in all the analysed samples of apo-ggNucb2 (Fig. 3) existed as monomers (apparent molecular mass, MMapp, of 51 kDa at the lowest protein concentration, which was in good agreement with the theoretical MM of 51.4 kDa). However, instead of one peak for the lowest apo-ggNucb2 concentration (Fig. 3A, B), two better-resolved peaks were detected with an increasing protein concentration (Fig. 3C-F), each of which could presumably be composed of one or more different components that undergo rapid and reversible association.

**Table 2:** Hydrodynamic properties of the Nucb2s.

Protein	Agent	c [mg/ml]	rmsd	s_(20,w)_ [S]	f/f_0_	R_h_ [nm]	MM_app_ [kDa]
ggNucb2	EDTA	0.5	0.005871	2.89	1.75	4.28	51 (100%)
		0.75	0.006157	2.46 3.04	1.75	3.99 4.43	41 (20%) 56 (80%)
		1.0	0.006470	2.42 3.08	1.76	3.97 4.48	40 (20%) 57 (80%)
	MgCl_2_	0.5	0.005721	3.09 5.69	1.74	4.42 6.00	57 (93%) 142 (7%)
		0.75	0.006160	2.78 3.31 5.61	1.74	4.20 4.58 5.97	49 (26%) 63 (67%) 139 (7%)
		1.0	0.006452	2.74 3.35 5.72	1.76	4.23 4.68 6.12	48 (21%) 65 (73%) 145 (6%)
	ZnCl_2_	0.5	0.005836	3.53 8.60	1.23	2.79 4.36	41 (88%) 156 (12%)
		0.75	0.006141	3.24 3.93 6.19 8.88	1.40	3.27 3.61 4.52 5.42	44 (37%) 59 (50%) 116 (3%) 200 (10%)
		1.0	0.006762	3.05 3.61 5.74 7.74	1.58	3.78 4.11 5.19 6.03	48 (35%) 61 (52%) 124 (5%) 194 (8%)
hsNucb2	EDTA	0.5	0.005929	2.50 3.37	2.07	5.21 6.06	55 (4%) 86 (96%)
		0.75	0.006217	2.42 3.38	2.04	5.02 5.94	51 (3%) 85 (97%)
		1.0	0.007139	2.41 3.39	2.07	5.15 6.10	52 (1%) 87 (99%)
	MgCl_2_	0.5	0.005852	2.76 3.55	1.74	4.23 4.81	49 (10%) 72 (90%)
		0.75	0.006220	2.71 3.56	1.83	4.49 5.15	51 (7%) 78 (93%)
		1.0	0.006917	2.70 3.56	1.89	4.72 5.43	54 (7%) 82 (93%)
	ZnCl_2_	0.5	0.006050	3.50 4.36 7.47	1.22	2.78 3.10 4.06	41 (18%) 57 (79%) 128 (3%)
		0.75	0.006101	3.17 4.03 6.37	1.47	3.53 3.98 5.00	47 (13%) 67 (85%) 135 (2%)
		1.0	0.006831	3.09 3.85	1.63	4.04 4.51	52 (14%) 73 (86%)

Numbers in brackets indicate the percentage of each fraction and are given considering 100% of the sum of the indicated main types of sedimenting species.

In the presence of 100 μM Zn^2+^ and 10 mM Mg^2+^, ggNucb2 was observed as a mixture of different species. A small fraction of ggNucb2 dimers (or trimers) was detected in the presence of Mg^2+^ (Table 2), suggesting that the binding of Mg^2+^ may influence the stability of the dimeric form. The most pronounced differences between the apo- and holo-states of ggNucb2 were observed after the addition of Zn^2+^ (Fig. 3A, C, E), where the major peaks appeared to be less prominent and considerably broader than those of the apo-protein samples. Zn^2+^-dependent association was not only manifested by ggNucb2s dimerization. We also observed the formation of higher-molecular-weight oligomers and an overall increase in the population of ggNucb2 conformations (Table 2). Moreover, the addition of Zn^2+^ up to a concentration of 300 μM caused a visible precipitation of ggNucb2 (each time when the protein concentration was ≤0.5 mg/ml), which we removed by extensive centrifugation prior to loading the cell (Fig. S1). The remaining soluble protein was still highly aggregated, indicating that Zn^2+^ induces oligomerization and the formation of large aggregates of ggNucb2s in solution.

**Fig. 4:**
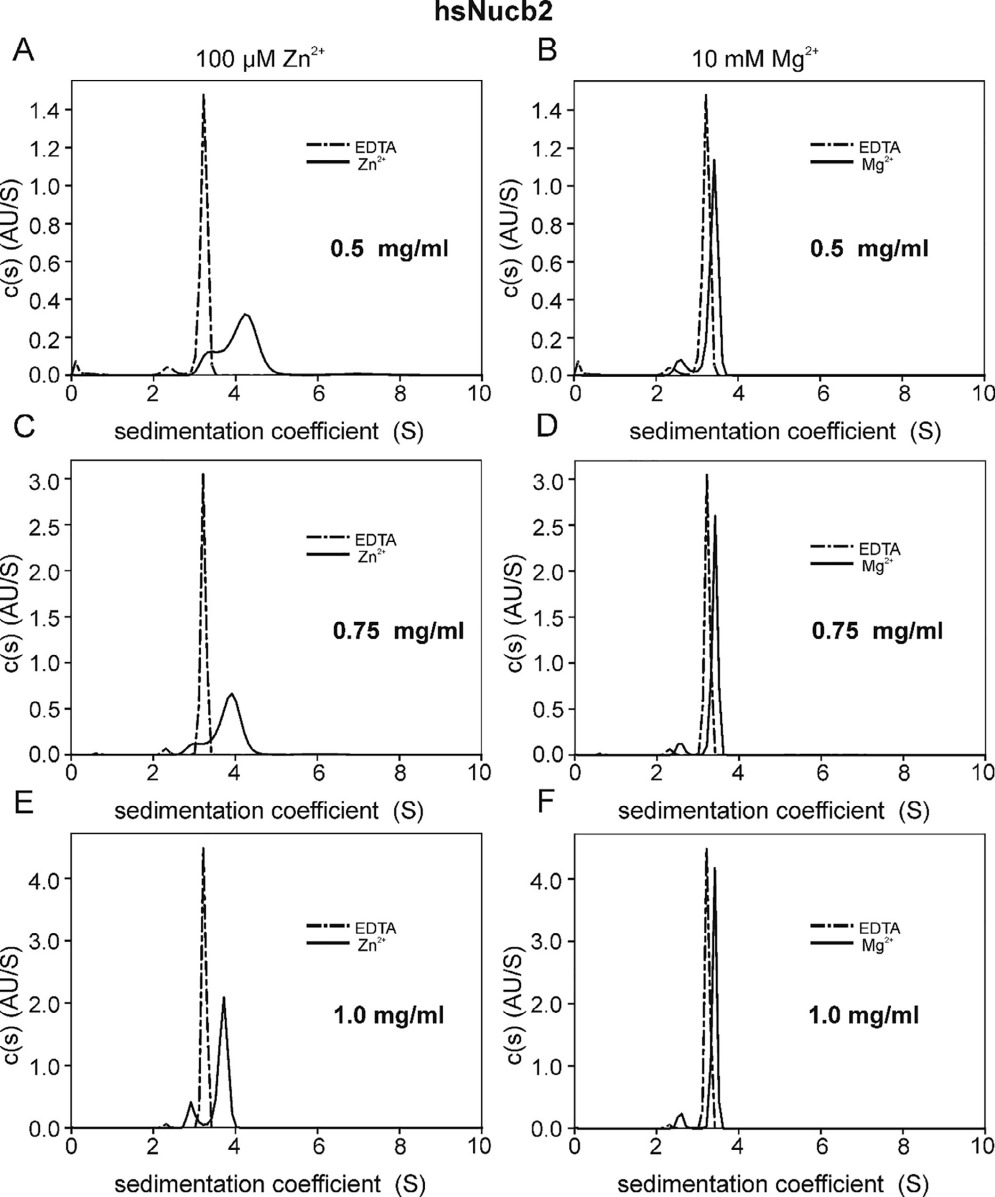
Size distribution characterization of hsNucb2 in the absence and presence of Zn^2+^ and Mg^2+^. The SV-AUC measurements were performed for three concentrations of hsNucb2: 0.5 (A, B), 0.75 (C, D) and 1.0 mg/ml (E, F) in the presence of 5 mM EDTA, 10 mM Mg^2+^ and 100 µM Zn^2+^, at 50 000 rpm and at 20 °C. Dash-dotted line – EDTA, solid line – appropriate metal ion.

In contrast, apo-hsNucb2 mostly existed in the dimeric state in solution (theoretical MM of the monomer: 47.7 kDa). A similar oligomerization state of hsNucb2 was observed in the presence of Mg^2+^ (Fig. 4B, D, F). On the other hand, in the presence of ZnCl_2_ (Fig. 4A, C, E), the amount of monomeric fractions increased (Table 2).

The AUC experiments revealed not only that the oligomerization state of the Nucb2s was changed in the presence of metal cations but also that the shape of both proteins changed. According to the collected data, apo-ggNucb2 turned out to be more compact than apo-hsNucb2 (frictional ratio – f/f_0_ of 1.76 and 2.07, respectively). An additional significant change in the f/f_0_ values was observed after Zn^2+^ binding. The f/f_0_ value decreased comparably for both proteins (Table 2), especially at a protein concentration of 0.5 mg/ml, indicating a conformational change to a more compact or more globular-like structure. In the presence of Mg^2+^, the f/f_0_ value of ggNucb2 remained the same compared to the apo-state of the protein, indicating no changes in the global conformation induced by this ligand. Simultaneously, for Mg^2+^-saturated hsNucb2, a small decrease in the f/f_0_ and R_h_ values compared to the apo state was observed. Additionally, the obtained results showed small concentration-dependent changes in the f/f_0_ values (the change from 1.74 to 1.89 with the concentration change ranging from 0.5 to 1.0 mg/ml) for holo-hsNucb2 after Mg^2+^ binding.

Based on the hydrodynamic properties determined by AUC, we can conclude that Nucb2s exist in solution as extended molecules, of which both the shape and the oligomerization state changed in the presence of metal cations, though in a different way for each of the homologues. The overall conformation of Mg^2+^-bound ggNucb2 seemed to be similar to the conformation of apo-ggNucb2 to a greater degree than that observed for hsNucb2 which, in turn, became more compact, especially at lower protein concentrations. On the other hand, observations made for Nucb2s saturated with Zn^2+^ suggested that not only oligomerization but also metal ion-driven aggregation processes could occur in the samples. Such aggregation may lead, in turn, to the formation of multiple extended protein conformations. This behaviour, to which ggNucb2 samples are considerably more vulnerable, is at the same time accompanied by protein precipitation at a specific Zn^2+^-to-protein molar ratio.

### 2.4 Thermodynamics of the interactions of Nucb2 with divalent metal ions

Fluorescence spectroscopy and AUC showed that Nucb2s in the presence of Zn^2+^ undergo tertiary and quaternary structural changes. On the other hand, Mg^2+^ has an effect on the oligomerization state of Nucb2s without significant secondary and tertiary structure changes. ITC measurements were performed to elucidate the thermodynamic parameters of Nucb2-Zn^2+^ and Nucb2-Mg^2+^ interactions.

**Fig. 5:**
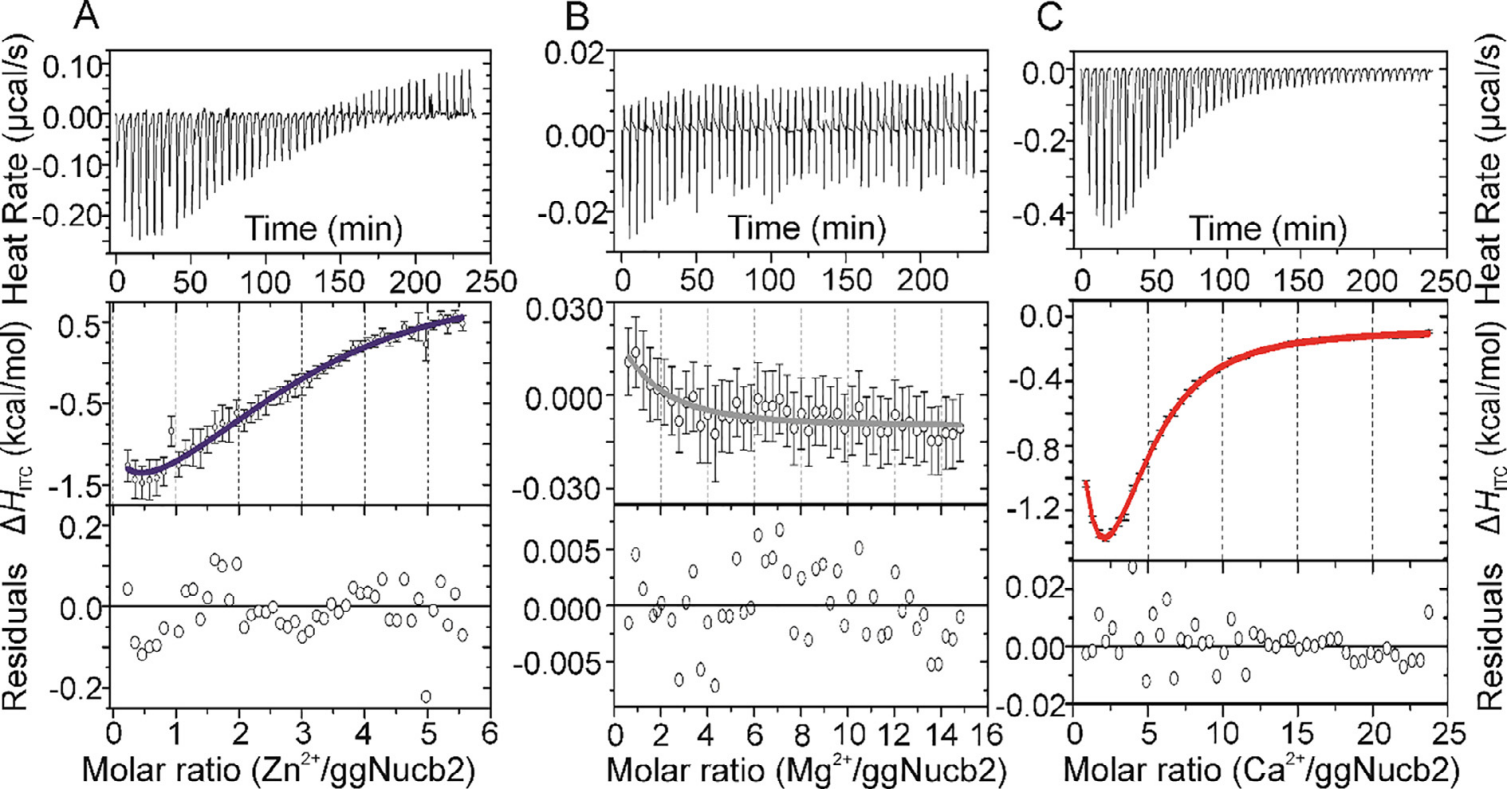
ITC analysis of Mg^2+^/Zn^2+^/ Ca^2+^ binding to ggNucb2. (A) ITC profiles for Zn^2+^ binding to ggNucb2, (B) ITC profiles for Mg^2+^ binding to ggNucb2, (C) ITC profiles for Ca^2+^ binding to ggNucb2. The top panels show the baseline-subtracted thermograms. The bottom panels represent the binding isotherm. All measurements were obtained in 20 mM HEPES and 150 mM NaCl (pH 7.5).

The first set of experiments focused on Zn^2+^ binding analysis. The binding of Zn^2+^ to ggNucb2 is represented by a complex thermogram that encompasses both exo- and endothermic processes (Fig. 5A). To further characterize additional equilibria, Zn^2+^ titration was performed with 1000 and 4000 µM titrant concentrations in a syringe (Fig. 5A, Fig. S2). The first experiment illuminated a gradually increasing exothermic process that may correspond with ligand-mediated dimerization. The second experiment, which employed the higher titrant concentration, demonstrated the contribution of the endothermic process that coincided with the first reaction, presenting higher absolute values of measured heats and of opposite sign. Nonetheless, a good fit for the two-binding-site model was generated. The obtained results showed that ggNucb2 preferentially formed monomer species and bound two Zn^2+^ ions, as the incompetent fraction stayed at 0. The first binding event occurred with a formation constant of approximately 190 µM and a binding enthalpy of −10 kcal/mol. The second binding event occurred with a much higher affinity of approximately 35 µM but with a small endothermic enthalpy of approximately +1 kcal/mol (Table 3). The corresponding entropic factor for the second event was very high compared with the entropic factor of the first binding with a +12 kcal/mol difference in TΔΔ*S*_2-1_. This result may suggest that the second process might be strongly influenced by the oligomeric state of the complex. Such conditions, however, effectively make fitted parameters questionable and uncertain.

**Table 3:** Thermodynamic parameters of Ca^2+^, Mg^2+^ and Zn^2+^ complexation with ggNucb2 derived from fitting data to the ABB model of interactions with two symmetrical binding sites and parameters of Mg^2+^ complexation derived from fitting to the AB model (see Materials and methods).

Parameter	ggNucb2 + Zn^2+^	ggNucb2 + Mg^2+^	ggNucb2 + Ca^2+^ (narrow range)	ggNucb2 + Ca^2+^ (broad range)
[Nucb2] (µM)	46.2	104.8	50	50
[M^2+^] (µM)	1000	6000	4000	4000
χ^2^	1.05	0.33	1.9	2.8
*K*_d1_ (µM)	188 ± 3	230 ± 32	190.02 ± 0.79	302.8 ± 0.9
Δ*H*_1_ (kcal/mol)	−10 ± 1	0.09 ± 0.11	−1.3 ± 0.2	−1.7 ± 0.1
TΔ*S*_1_ (kcal/mol)	−4.92	5.05	3.78	3.11
*K*_d2_ (µM)	34.38 ± 1.87	–	73.2 ± 0.7	60.6 ± 0.4
Δ*H*_2_ (kcal/mol)	1.16 ± 0.48	–	−8.1 ± 0.3	−9.6 ± 0.8
TΔ*S*_2_ (kcal/mol)	7.25	–	−2.46	−3.85
Inc. Nucb2 fraction	0	0	0.00 ± 0.05	0.00 ± 0.10

Nucb2 – nucleobindin 2; M^2+^ – metal ion (Ca^2+^, Zn^2+^ or Mg^2+^); χ^2^ – chi square, *K*_d1/d2_ – dissociation constant for the first or the second binding site; Δ*H*_1/2_ – enthalpy of the complex formation for the first or the second binding site; TΔ*S*_1/2_ – entropic contribution for the first or the second binding event; Inc. Nucb2 fraction – incompetent protein fraction.

**Fig. 6:**
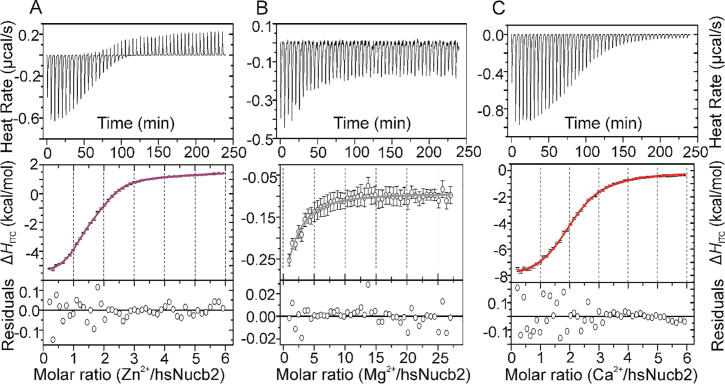
ITC analysis of Mg^2+^/Zn^2+^/Ca^2+^ binding to hsNucb2. (A) ITC profiles for Zn^2+^ binding to hsNucb2, (B) ITC profiles for Mg^2+^ binding to hsNucb2, (C) ITC profiles for Ca^2+^ binding to hsNucb2. The top panels show the baseline-subtracted thermograms. The bottom panels represent the binding isotherm. All measurements were obtained in 20 mM HEPES and 150 mM NaCl (pH 7.5).

Zn^2+^ binding to hsNucb2 (Fig. 6A) is represented by an exothermic process with threefold higher negative heats (in absolute values) than Zn^2+^-titrated ggNucb2. Additionally, the hsNucb2 titration is represented by a monophasic isotherm with a single inflection point and a higher slope compared with the analogous titration of ggNucb2. The results were best fitted to the two-binding-site model with binding enthalpies of approximately −11 kcal/mol and −8 kcal/mol, respectively (Table 4). The calculated Δ*G*_ITC_ values were similar for both binding events, which seemed to be the consequence of the varied entropic contribution, with the second site having a greater entropic impact than the other. These results suggested that the two binding sites may be differentiated in terms of ligands and may therefore be structurally and functionally different. An incompetent fraction of hsNucb2 was best fitted to approximately 0.5, suggesting that the protein in its apo form is predominately in a dimeric conformation (see Table 4).

**Table 4:** Thermodynamic parameters of Ca^2+^ and Zn^2+^ complexation with hsNucb2 derived from fitting data to the ABB model of interactions with two symmetrical binding sites and parameters of Mg^2+^ complexation derived from fitting to the AB model (see Materials and methods).

Parameter	hsNucb2 + Zn^2+^	hsNucb2 + Mg^2+^	hsNucb2 + Ca^2+^
[Nucb2] (µM)	75.2	65.9	75.2
[M^2+^] (µM)	1500	6000	1500
χ^2^	0.42	0.71	4.8
*K*_d1_ (µM)	23.9 ± 0.2	203.21 ± 0.01	16.8 ± 0.2
Δ*H*_1_ (kcal/mol)	−11.0 ± 0.3	−0.85 ± 0.02	−9.1 ± 0.2
TΔ*S*_1_ (kcal/mol)	−6.04	4.186	−2.61
*K*_d2_ (µM)	14.3 ± 0.7	–	19.8 ± 0.2
Δ*H*_2_ (kcal/mol)	−8.4 ± 0.6	–	−10.4 ± 0.3
TΔ*S*_2_ (kcal/mol)	−1.76	–	−3.98
Inc. Nucb2 fraction	0.57 ± 0.01	0.00 ± 0.01	0.48 ± 0.07

Nucb2 – nucleobindin 2; M^2+^ – metal ion (Ca^2+^, Zn^2+^ or Mg^2+^); χ^2^ – chi square, *K*_d1/d2_ – dissociation constant for the first or the second binding site; Δ*H*_1/2_ – enthalpy of the complex formation for the first or the second binding site; TΔ*S*_1/2_ – entropic contribution for the first or the second binding event; Inc. Nucb2 fraction – incompetent protein fraction.

Next, we focused on the thermodynamics of Mg^2+^ binding to both Nucb2s. Mg^2+^ titration of Nucb2s showed that one Mg^2+^ ion bound to one Nucb2 protein molecule (Fig. 5B and Fig. 6B) with a relatively high affinity (considering the millimolar buffering capacity of cells towards Mg^2+^ [60]) of approximately 200 µM, and the process was entropy-driven. Importantly, the presented heats were extremely small, increasing the uncertainty of the fitted parameters, especially ggNucb2. Mg^2+^ binding to ggNucb2 seemed to be an endothermic reaction; however, its heat rate was comparable to the dilution heat rate with a positive heat value, which almost entirely suppressed the resulting heat. Nevertheless, the presented results showed that both proteins differed significantly in terms of their qualitative assessment of Mg^2+^ binding, suggesting that the metal binding sites of both orthologues were not similar.

A previous study (see Ref. 49) revealed that both Nucb2s underwent Ca^2+^-dependent structural modulation. HDX-MS showed that Ca^2+^ induced the compaction of two EF-hands of Nucb2s [49]. We also utilized ITC to determine the thermodynamic parameters of the Ca^2+^-Nucb2s interaction. Ca^2+^ binding to ggNucb2 is represented by a biphasic process with increasing exothermic heats at the beginning of titration (Fig. 5C). The exothermic process is described by the two-binding-site model, with substantially different affinities being observed for both sites. *K*_d2_ is approximately 5 times lower than *K*_d1._ Moreover, the first binding event was determined to be more entropy-driven, with the second being more enthalpy-driven (Table 3). Moreover, the incompetent protein fraction was negligible, which suggests that ggNucb2 in its apo form is predominantly monomeric. Nonetheless, gradual increases in the exothermic process, clearly distinguishable for ggNucb2 titrated with Ca^2+^, may be caused by the ligand-mediated association of protein monomers into dimers or higher oligomeric forms. Fitting accounting for this process suggested that metal ions shift the equilibrium of oligomerization towards dimers and that the process is cooperative. However, on the basis of the presented results alone, we cannot determine the values of the binding constants and cooperativity index values with certainty, as additional experimental conditions are necessary to obtain these parameters with precision (Table S1).

The binding of Ca^2+^ to the hsNucb2 protein (Fig. 6C) is very different from the binding of Ca^2+^ to ggNucb2. The binding process is represented by a monophasic isotherm, with the experimental heats being considerably more pronounced. In this analysis, the exothermic process was best fitted to the ABB model accounting for two symmetrical sites with a binding enthalpy for both sites of approximately −10 kcal/mol. The results show that two Ca^2+^ ions bind macroscopically identical to both sites of hsNucb2, contrary to ggNucb2. However, the fitted parameter of the incompetent protein fraction of approximately 0.5 suggested that 50% of protein molecules were not involved in the binding processes as individuals, indicating that Ca^2+^ ions are bound by a dimeric unit of hsNucb2 (Table 4).

Nucb2s are in monomer-dimer equilibrium or even monomer-oligomer equilibrium in the case of the ggNucb2 homologue. This property makes the analysis of ITC isotherms challenging, as metal ion titration would be initiated into unstable oligomeric species. The clear differences in isotherm shapes of Zn^2+^ and Ca^2+^ binding to Nucb2s, as well as varied entropic factors, suggest that hsNucb2 is preferentially dimeric in the apo state, which stands in contrast to ggNucb2, which presents a significant fraction of monomer before metal ion addition, which is in keeping with the SV-AUC results.

### 2.5 Enhanced dynamics of Nucb2s showed by HDX-MS

Fluorescence spectroscopy showed that the structure of both Nucb2s undergoes compaction in the presence of Zn^2+^. The AUC results revealed that Nucb2s have a compact shape and exist as oligomeric species in the presence of Zn^2+^. The ITC measurements confirmed that Nucb2s are Mg^2+^/Zn^2+^-binding proteins. To investigate the impact of Zn^2+^ and Mg^2+^ binding on the structure and conformational dynamics of Nucb2s and to identify the potential residues or regions involved in these binding events, we utilized HDX-MS. The HDX-MS technique is based on the monitoring of the exchange of amide hydrogen to deuterium. The rate of these exchanges depends on the conformational flexibility of the protein’s backbone, solvent accessibility, and hydrogen bonding status [61], [62]. Amide hydrogens involved in bonding and located within the secondary and tertiary structural elements exchange slowly. In contrast, the exchange of amide hydrogens exposed to the solvent and not engaged in ordered structural elements is rapid [63], [64]. HDX-MS is currently used to detect IDRs in proteins due to its ability to differentiate between protein regions of diverse deuterium exchange protection [65].

**Fig. 7:**
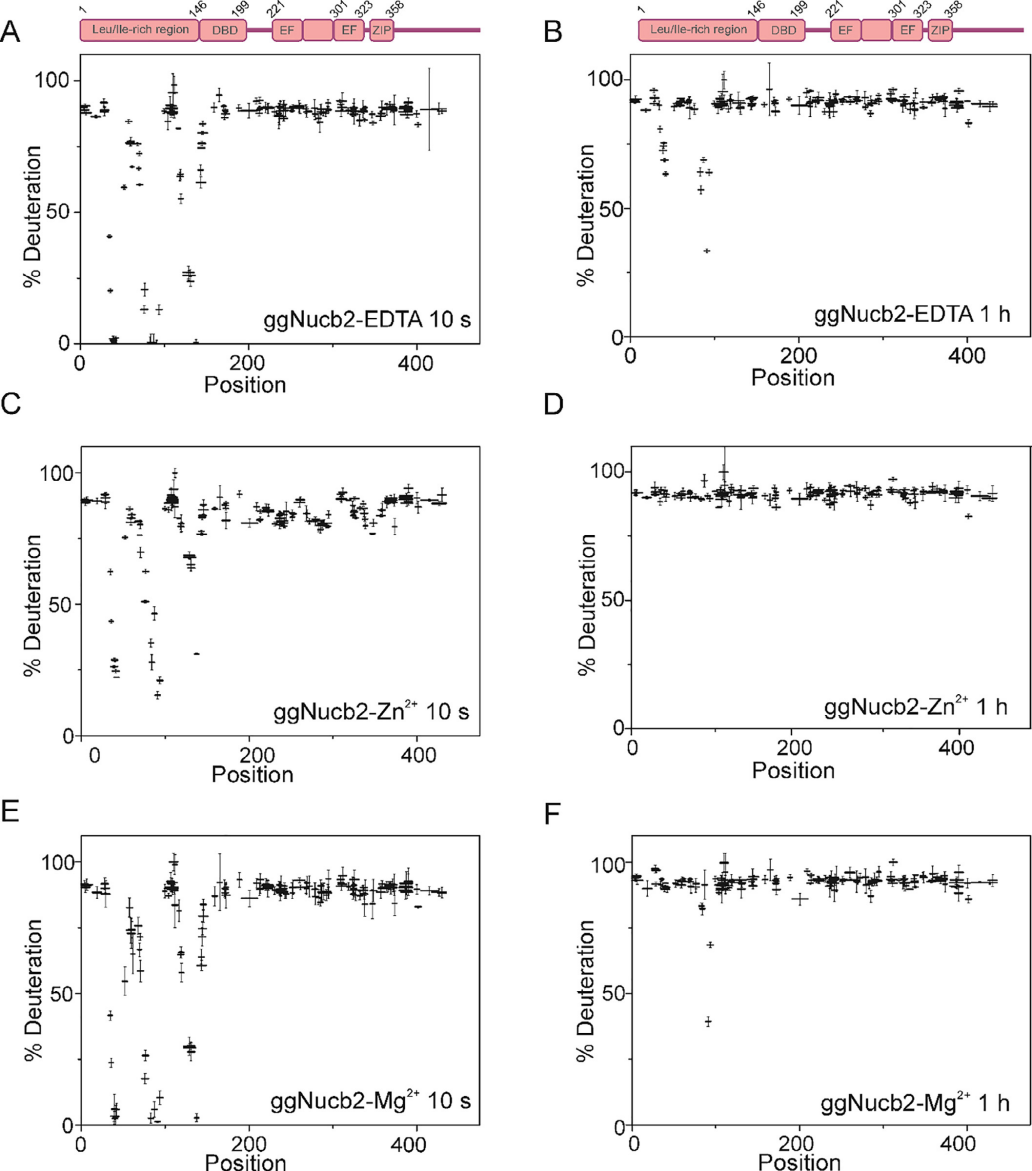
Percentage of deuteration in peptides from ggNucb2 in the absence and presence of 100 µM Zn^2+^ and 10 mM Mg^2+^. The graphs represent HDX patterns after 10 s (A, C, E) and 1 h (B, D, F) incubation with D_2_O. The error bars represent the standard deviations of three independent experiments.

**Fig. 8:**
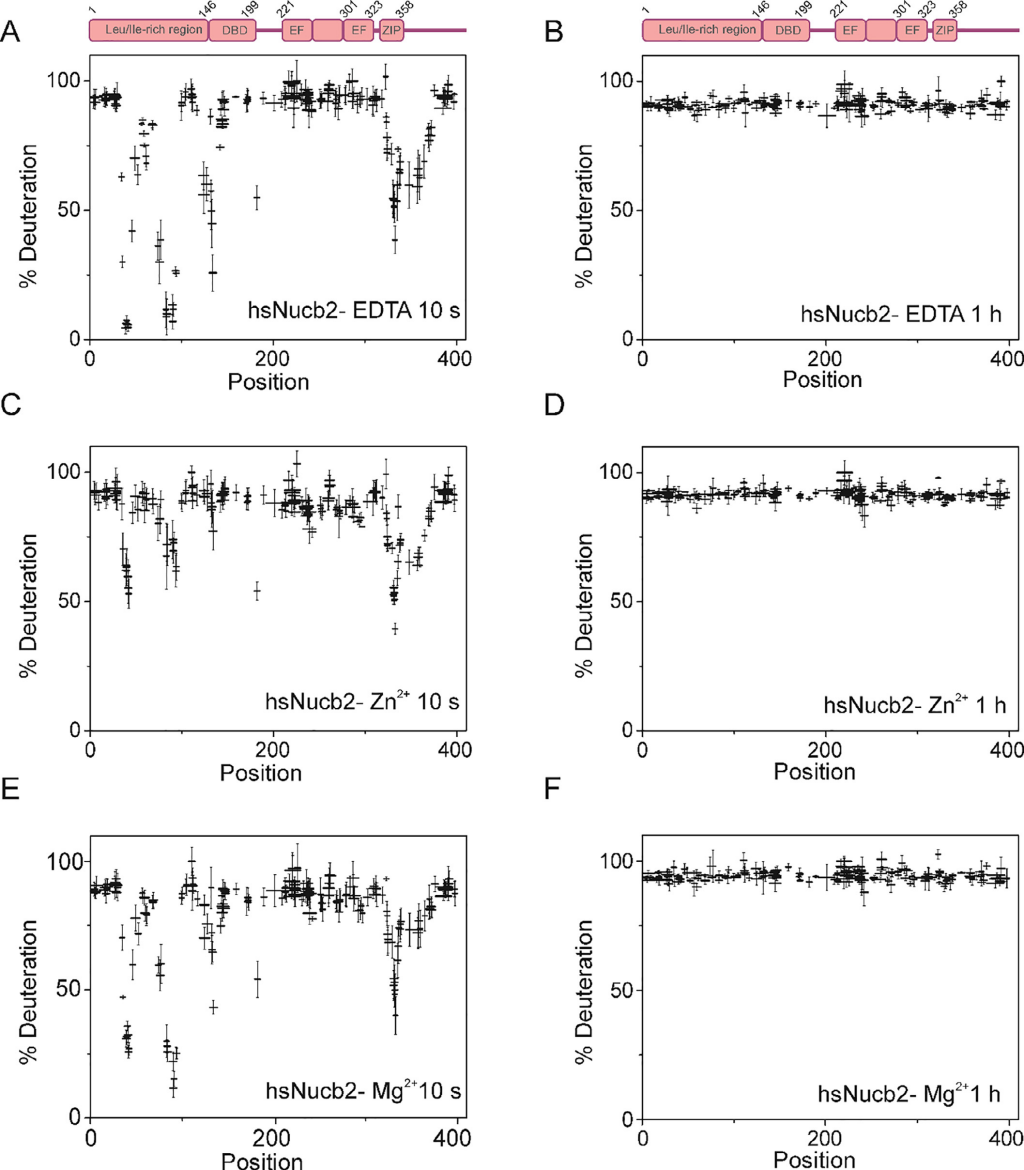
Percentage of deuteration in peptides from hsNucb2 in the absence and presence of 100 µM Zn^2+^ and 10 mM Mg^2+^. The graphs represent HDX patterns after 10 s (A, C, E) and 1 h (B, D, F) incubation with D_2_O. The error bars represent the standard deviations of three independent experiments.

Before each experiment, both proteins were digested using an immobilized Nepenthesin-2 column to produce a peptide map enabling the best coverage of the protein sequence. For HDX-MS analysis, 161 peptides of ggNucb2 covering 92.4% of the sequence and 200 peptides of hsNucb2 covering 95.0% of the sequence were selected. We followed the hydrogen to deuterium exchange of Nucb2s over a time course of 10 s to 1 h. The structure of Nucb2s (Fig. 7A and Fig. 8A) was first studied to obtain wide structural information on ligand-free protein molecules. The C-terminal halves of both proteins were characterized by fast HDX (Fig. 7A and Fig. 8A), suggesting that they have high conformational flexibility and are structurally dynamic. In contrast, the N-terminal halves of Nucb2s were characterized by a mosaic-like structure consisting of regions with rapid or slow HDX rates. Notably, the level of deuterium uptake observed for peptides encompassing amino acid residues 33–45, 80–97 and 135–140 in the ggNucb2 sequence (Fig. 7B) did not reach the maximal value even after 1 h. Such structural protection indicates that these regions form a very stable core structure. The results obtained in this analysis were in keeping with previously presented findings for both His-tagged apo-Nucb2 proteins (see Ref. 49).

To characterize the Zn^2+^-induced conformational rearrangements of Nucb2s, we examined the HDX rate in the presence of these metal ions. In the presence of 100 µM Zn^2+^ (Fig. 7C and Fig. 8C), changes in deuterium uptake only occurred at the N-terminal half of the Nucb2s. Three regions (peptides 32–55, 72–94 and 115–149) of ggNucb2 (Fig. 7C and D) and three regions of hsNucb2 (peptides 33–55, 71–94 and 129–138) (Fig. 8C and D) had higher deuterium uptake after the addition of Zn^2+^, indicating the increased solvent accessibility of these regions. Notably, a change in the HDX level of Nucb2s occurred in the region of the putative Zn^2+^-binding site HFREX_n_H [48]. For hsNucb2, the major HDX differences between apo- and Zn^2+^-hsNucb2 were prominent at early time points: 10 s and 1 min (data not shown). After 10 min of exchange, the difference was not detected (data not shown). The increase in solvent exposure within 10 s was higher in holo-hsNucb2 than that observed for Zn^2+^-ggNucb2. However, differences in the conformational flexibility and solvent exposure between Zn^2+^- and EDTA-ggNucb2 were still observed for peptides 33–45, 80–97 and 135–140 even after a one-hour exchange (Fig. 7B and D).

Next, we investigated the effect of Mg^2+^ on the solvent accessibility of Nucb2 proteins. The HDX-MS experiment in the presence of 10 mM Mg^2+^ was performed under the same conditions as in the presence of 5 mM EDTA and 100 µM Zn^2+^. The HDX pattern of ggNucb2 in the presence of EDTA and Mg^2+^ was notably similar (Fig. 7E and F), which was in keeping with the CD and fluorescence spectra results. However, the ITC revealed that ggNucb2 bound one Mg^2+^. This result indicated that the binding of Mg^2+^ by ggNucb2 might not induce significant structural rearrangements of the protein structure. Notably, the deuterium uptake level of hsNucb2 (Fig. 8E and F) was slightly increased in the presence of Mg^2+^. Changes in deuterium incorporation were visible in the N-terminal half of hsNucb2 (peptides 33–49, 71–94 and 129–138), which was also observed for Zn^2+^. However, the increase in deuterium incorporation in the presence of Mg^2+^ was less pronounced. Overall, the presence of Zn^2+^ ions led to increased solvent exposure of the N-terminal half of the Nucb2s, which was probably due to destabilization of this protein part. Furthermore, Zn^2+^-dependent structural changes were more significant for ggNucb2. The presence of Mg^2+^ was only determined to have an impact on the solvent accessibility of hsNucb2. Similar to Zn^2+^, the addition of Mg^2+^ led to increased flexibility of the N-terminal part of hsNucb2. However, the change in the HDX level in the presence of Mg^2+^ was smaller than that in the presence of Zn^2+^.

### 2.6 Change in the structural parameters of Nucb2 characterized by SAXS

**Fig. 9:**
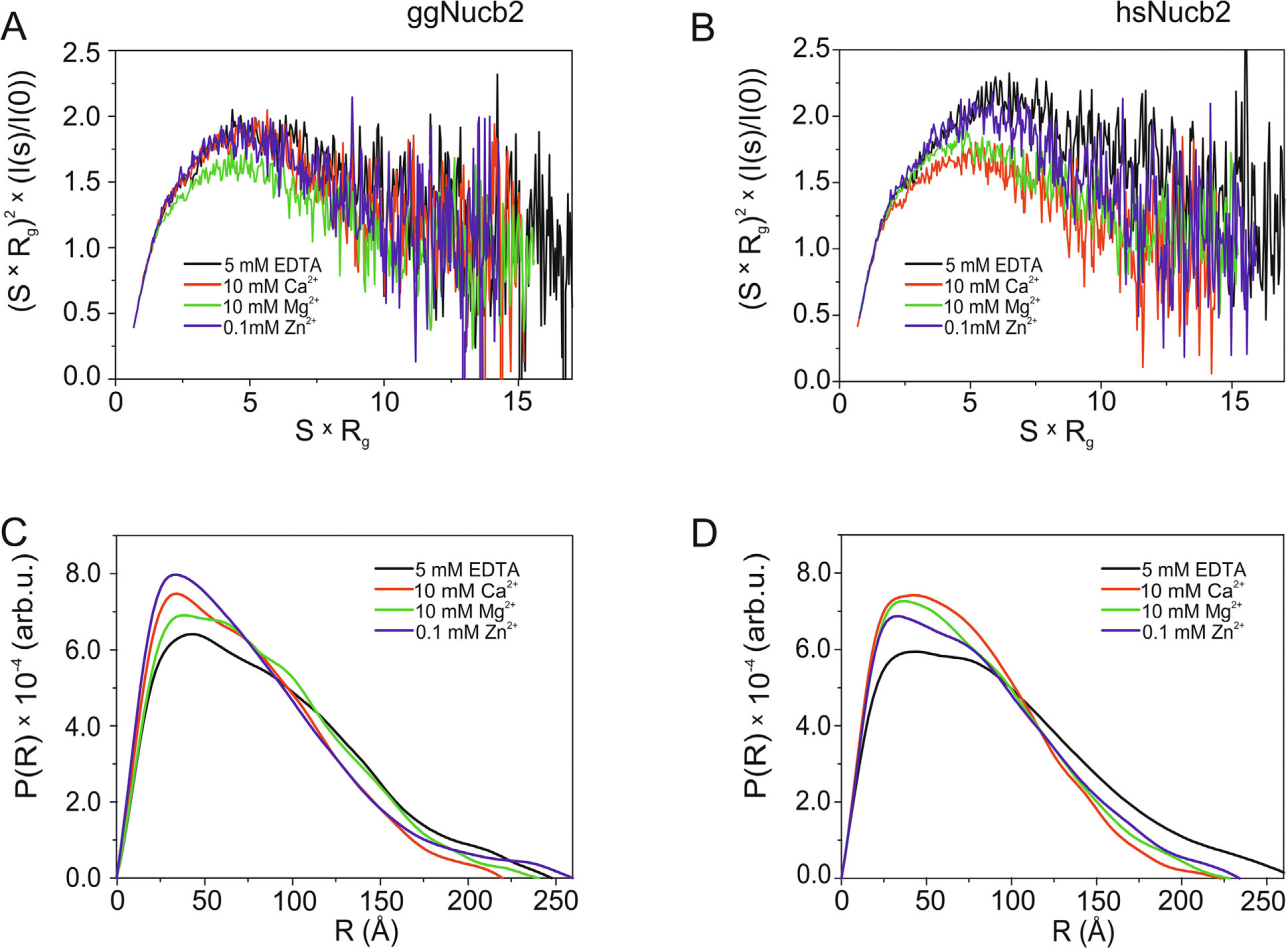
SAXS analysis of Nucb2s. Dimensionless Kratky plot of ggNucb2 (A) and hsNucb2 (B). The pair distribution function *p*(r) of ggNucb2 (C) and hsNucb2 (D). SAXS measurements were carried out on Nucb2 in the presence of 5 mM EDTA, 10 mM Ca^2+^, 10 mM Mg^2+^ and 100 µM Zn^2+^.

Trp fluorescence spectra analysis and AUC data showed that in the presence of Zn^2+^, Nucb2s undergo compaction and oligomerization. Moreover, HDX-MS revealed solvent exposure at the N-terminal half of the Nucb2s. To further investigate the structural changes of Nucb2s induced by metal ions, we employed SAXS, which is a powerful biophysical technique that provides information regarding the overall shape and size of molecules in solution under various conditions [66], [67]. This method is frequently used to study different IDPs [68]. We performed SAXS analyses of both Nucb2s in the absence and presence of Zn^2+^, Mg^2+^ and Ca^2+^. The scattering data of both Nucb2s in the absence and presence of metal ions were qualitatively assessed by means of a Kratky plot (Fig. 9A and B). All SAXS curves of Nucb2s showed a broad, asymmetric peak and a plateau followed by a further decrease at large S values. This behaviour is characteristic of partially disordered proteins and multidomain proteins [69].

The radius of gyration, R_g_, which is determined from the Guinier plot’s slope, is frequently used to quantify the average size of macromolecules [70]. The R_g_ is higher for partly extended or even fully disordered proteins than for globular particles [71]. The R_g_ values for ggNucb2 and hsNucb2 in the presence of EDTA were calculated to be 66.5 ± 1.9 Å and 67.6 ± 2.3 Å, respectively (Table 5). Notably, in the presence of metal ions (Zn^2+^, Mg^2+^ and Ca^2+^), the R_g_ for both Nucb2s decreased, suggesting the compaction of the Nucb2 molecules. The change in R_g_ was observed to be most significant for Zn^2+^-Nucb2s. The pair distance distribution function, *p*(r) (which is a histogram of all the distances within the protein molecule [68]), was calculated for all the apo- and holo-forms of Nucb2 by indirect Fourier transform of the scattering intensity using the GNOM program [72]. The *p*(r) functions for all forms of Nucb2 were determined to be asymmetric and displayed extended tails (see examples in Fig. 9C and D), which is typical for proteins with an elongated shape. This property was also indicated by the large maximum particle dimension (D_max_) values of all the Nucb2 forms. However, in the presence of analysed metal cations, the D_max_ values decreased for both Nucb2. The only exception was observed for Zn^2+^-ggNucb2, where binding of Zn^2+^ led to an increase in the D_max_ value (Table 5). Notably, the characteristic shape of the Nucb2s’ *p*(r) function indicated the presence of two species in solution. Therefore, we also applied the obtained experimental SAXS data to determine the molecular mass and the oligomeric state of both proteins. The method uses the extrapolated intensity I(0) on a relative scale, and its further comparison with I(0) from a standard protein with known molecular mass (here BSA) was chosen for this purpose. The analysis revealed that ggNucb2 in apo- and all holo-forms, as well as hsNucb2 in the presence of Zn^2+^, existed in the solution as a mixture of monomers and dimers, while the dominant form of apo- and Mg^2+^/Ca^2+^-hsNucb2 in solution was a dimer. These results were in keeping with the presented AUC data. The differences in *p*(r) shape through the different forms of both Nucb2s may be attributable to structural reorganization within the molecular species.

**Table 5:** Structural parameters of Nucb2s in the absence and presence of different metal ions determined from SAXS data.

Agent	R_g_ [Å]	D_max_ [Å]	MM [kDa]
ggNucb2			
5 mM EDTA	66.5 ± 1.9	248	82.75
10 mM Mg^2+^	60.5 ± 1.6	240	85.91
100 µM Zn^2+^	55.29 ± 1.68	260	67.68
10 mM Ca^2+^	58.85 ± 1.19	220	75.39

hsNucb2			
5 mM EDTA	67.62 ± 2.27	268	91.87
10 mM Mg^2+^	58.92 ± 1.76	228	92.92
100 µM Zn^2+^	61.64 ± 1.40	234	82.40
10 mM Ca^2+^	55.73 ± 1.13	223	93.62

### 2.7 Metal-ion-dependent morphology of Nucb2 aggregates

During CD and ITC experiments, we observed that ggNucb2 underwent precipitation upon a higher concentration of Zn^2+^. Additionally, the AUC showed that Nucb2s in the presence of Zn^2+^ existed in the solution as high-molecular-weight assemblies and that precipitation of ggNucb2 was induced by a Zn^2+^ concentration of ≥300 µM. To visualize the morphology of higher-molecular-weight aggregates of ggNucb2, we monitored the supernatant and insoluble precipitate of 0.5 mg/ml ggNucb2 obtained after AUC by transmission electron microscopy (TEM). TEM images revealed that ggNucb2 precipitates were spherical aggregates and irregular assemblies of amorphous aggregates (see Fig. S3), distributed evenly. On the other hand, ggNucb2 isolated from the supernatant fraction consisted of spherical aggregates with an average diameter of 24 ± 4 nm and elongated particles with an average diameter of 32 ± 14 nm (Fig. S3).

**Fig. 10:**
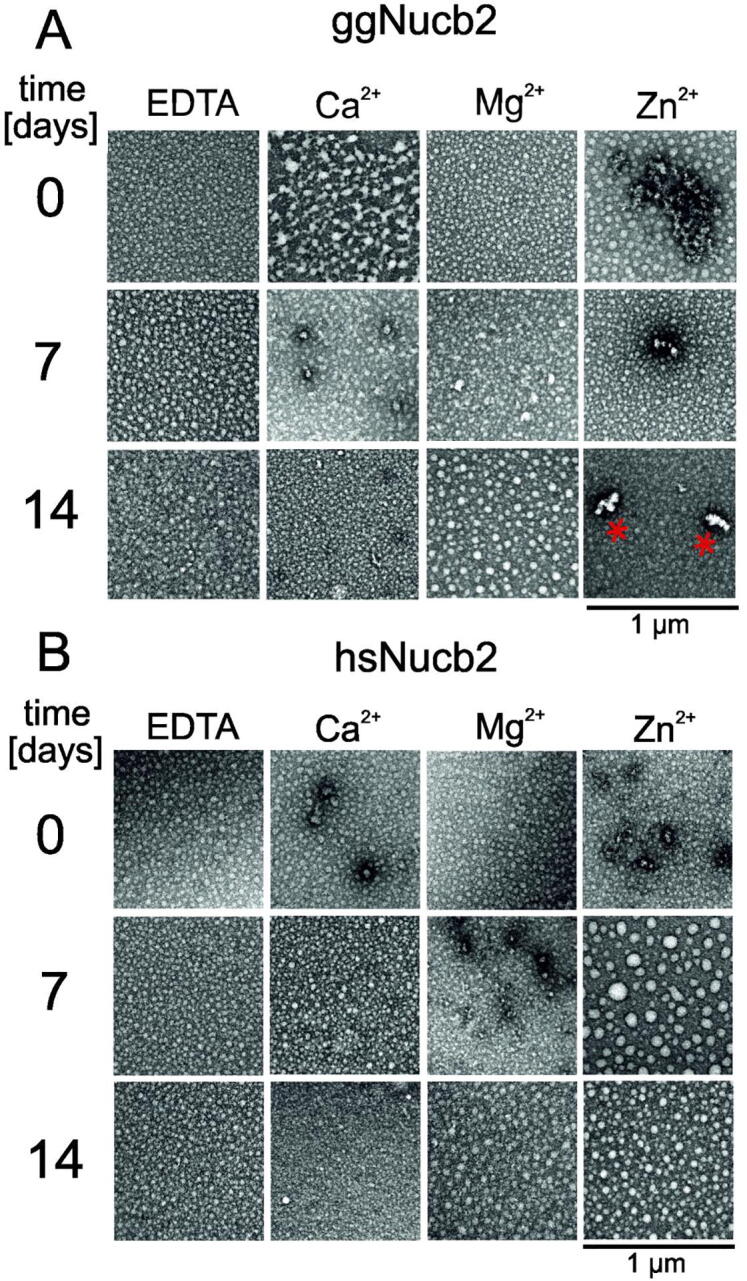
Transmission electron microscopy (TEM) micrographs of Nucb2 aggregate morphology in the presence of divalent metal ions at different time points. (A) Representative TEM images of ggNucb2 aggregates generated in the absence and presence of 10 mM Ca^2+^, 10 mM Mg^2+^ and 300 µM Zn^2+^ in the 0–14-day periods. (B) Representative TEM images of hsNucb2 aggregates generated in the absence or in the presence of 10 mM Ca^2+^, 10 mM Mg^2+^ and 300 µM Zn^2+^ in the 0–14-day periods. The red asterisks indicate the elongated particles. Scale bars represent 1 µm. (For interpretation of the references to colour in this figure legend, the reader is referred to the web version of this article.)

To further analyse the morphology of Nucb2 aggregates, TEM images were obtained at different time points (0–2 weeks) for Nucb2s incubated in the absence and presence of 300 µM Zn^2+^, 10 mM Mg^2+^ and 10 mM Ca^2+^. We chose 300 µM Zn^2+^ as a critical concentration, which induced the greatest oligomerization changes revealed by SV-AUC and led to protein precipitation when the protein concentration was maintained at or below 0.5 mg/ml. In the absence of metal ions, both Nucb2s formed spherical aggregates that were similar in size even after a 2-week incubation period (Fig. 10A and B). The heterogeneous mixture of Nucb2 round particles was observed at 10 mM Ca^2+^. Notably, the size of Nucb2 aggregates in the presence of Ca^2+^ decreased over time. A similar tendency was seen for Mg^2+^-hsNucb2 particles. In contrast, the size of ggNucb2 spherical aggregates in the presence of Mg^2+^ increased throughout the two-week incubation period. However, the most significant alterations in the morphology of Nucb2 aggregates were observed in the presence of Zn^2+^. The incubation of ggNucb2 with 300 µM Zn^2+^ resulted in spherical aggregates and elongated particles (Fig. 10A), even after two weeks. In contrast, in the presence of Zn^2+^, hsNucb2 formed well-dispersed aggregates (see Fig. 10B). These results were consistent with the previously described AUC data. Zn^2+^ bound to Nucb2s, inducing the formation of higher-order aggregates. This effect was more visible for ggNucb2.

## 3 Discussion

The binding of metal ions impacts the structure of proteins which, in turn, may be engaged in the regulation of their functions [73], [74]. Metal ion binding might lead to disorder-to-order transition [75], destabilization of the structure [76], oligomerization [77] and even aggregation [78] of the protein. Changes in the protein structure in the presence of various metal ions might facilitate the adaptation to specific protein functions. Among all metal ions, Ca^2+^, Zn^2+^, and Mg^2+^ are the most relevant for modulating protein function [79]. Previous research [49] revealed that Ca^2+^ has a strong impact on the structure of Nucb2s. The interaction between Ca^2+^ and Nucb2s resulted in a disorder-to-order transition and dimerization of both proteins [49]. In the structure of Nucb2s, two EF-hand domains are present, which are responsible for Ca^2+^/Mg^2+^ binding [6] and one putative motif of Zn^2+^-binding [48]. In this study, we determined the thermodynamic properties of Nucb2s interacting with Mg^2+^ and Zn^2+^, as well as their influence on the structure of Nucb2s. The far-UV CD indicated that Zn^2+^ slightly modulated the secondary structure of Nucb2s. HDX-MS analysis revealed that upon the addition of Zn^2+^, the solvent accessibility of regions of the N-terminal half (containing nesfatin-1 and -2) of Nucb2s is changed (increased), leading to a decrease in the stability of the protein molecule. The structural dynamics change of ggNucb2s in the presence of Zn^2+^ was found to be even more pronounced. On the other hand, Trp emission spectra showed that Nucb2s upon Zn^2+^ binding undergo structural compaction, which is in keeping with limited proteolysis (see Figs. S5 and S6), SV-AUC study and SAXS data. Both of the Trp residues of Nucb2s are located at the C-terminal half, i.e., nesfatin-3. These results suggest that Zn^2+^ might also affect the structure of nesfatin-3. Therefore, we conclude that for both homologues, Zn^2+^ binding manifests itself by significant conformational changes, which seem to also be consequences of changes in the secondary structure of both protein molecules. In contrast, Mg^2+^ binding does not seem to be followed by ggNucb2 secondary structure alternation according to CD and fluorescence data, indicating a different binding mechanism for this ligand. Mg^2+^ induces, however, a slight increase of α-helical content of hsNucb2. We also did not observe compaction of Mg^2+^-saturated ggNucb2 with only a minor compaction of the Mg^2+^-hsNucb2 molecule. Mg^2+^ binding may, however, change the monomer-oligomer equilibrium, especially for ggNucb2. Additionally, HDX-MS showed that the presence of Mg^2+^ slightly affects conformational flexibility but only affects hsNucb2. Notably, both Zn^2+^/Mg^2+^ ions affect the oligomeric state of Nucb2s. Apo-ggNucb2 behaves as a monomer in the solution. ggNucb2 in the presence of Mg^2+^ consists of monomeric species and a low amount of dimeric species. These results suggest that the addition of Mg^2+^ changes the equilibrium between the monomeric and dimeric forms of ggNucb2, leading to dimeric form stabilization. In contrast, hsNucb2, in its apo- and holo-(Mg^2+^) form, exists primarily in a dimeric form. Surprisingly, Zn^2+^ addition results in the appearance of high-molecular-weight assemblies of Nucb2s, which are more evident for ggNucb2. TEM images revealed that the addition of Zn^2+^ resulted in the formation of characteristic aggregates that were well dispersed and elongated for hsNucb2 and ggNucb2, respectively. Additionally, a high concentration of Zn^2+^ (≥300 µM) induces the precipitation of ggNucb2 during the CD, fluorescence and SV-AUC experiments. TEM analysis visualized the appearance of characteristic assemblies of amorphous aggregates in the ggNucb2 precipitate and elongated particles in the isolated supernatant form of ggNucb2. Notably, similar irregular assemblies were observed for the intermediate filament protein vimentin in the presence of Zn^2+^ [80]. These vimentin fibrils are not able to mediate filament formation [80]. Vimentin is expressed in mesenchymal cells and participates in such processes as cell division, migration and signalling [81], [82], [83]. Vimentin is also known as a marker and agent of the epithelial to mesenchymal transition (EMT) process [84], [85]. Surprisingly, previous research suggested that Nucb2 is involved in EMT [41], [86]. This Zn^2+^-dependent structural alteration of Nucb2 molecules might be connected with the role of Nucb2 in the tumorigenesis process. However, the precipitation and formation of similar aggregates in the presence of elevated Zn^2+^ concentrations were also observed, e.g., neuronal calcium sensor-1 (NCS-1) protein [87] and insulin [88]. Brain cells [89] and β cells in the pancreas [24] are characterized by high levels of Zn^2+^. Zn^2+^ participates in both brain physiology and pathology, and it is essential to maintain normal brain activity and proper protein function [90]. On the other hand, alterations in Zn^2+^ levels are implicated in central nervous system disorders, such as neurodegenerative diseases [91], [92] and mental dysfunction, e.g., depression [28] and schizophrenia [93]. Moreover, Zn^2+^ ions are implicated in β-cell insulin formation, storage and secretion [94], [95]. Notably, an elevated expression level of Nucb2 was found in the central nervous system, i.e., the hypothalamus and pituitary gland [38], [96], and in pancreatic β cells [45]. Additionally, it was shown that Nucb2 and nesfatin-1 promote insulin secretion, which suggests the antidiabetic properties of these proteins [97]. The physiological importance of Zn^2+^ for Nucb2 function has not been determined. Taken together, our results suggest that Zn^2+^ binding to the Nucb2 molecule leads at first to the solvent exposure of peptides at the N-terminal half of Nucb2s, probably enabling structural changes at the C-terminal half of Nucb2s and the formation of oligomeric species of both homologues. These formed oligomers/aggregates might constitute physiological or pathological forms of Nucb2s. The question of whether this self-association and/or precipitation would be physiologically relevant merits further investigation.

To gain insights into the binding of Zn^2+^/Mg^2+^ to Nucb2s, we used ITC. The Mg^2+^-Nucb2 interaction for both homologues appeared to be highly similar. ITC demonstrated the existence of one binding site for Mg^2+^ with high affinity. On the other hand, the binding of Zn^2+^ by both Nucb2s was found to be more complex. Although Zn^2+^ binding events were predominately exothermic processes, thermograms involved a small fraction of endothermic heats. Moreover, the interaction of ggNucb2 with Zn^2+^ exhibited significantly more complicated behaviour than hsNucb2. At a lower concentration of Zn^2+^, the exothermic reaction was determined to coincide with an endothermic process, as demonstrated by gradually increasing negative heats at the first several injection points. In contrast, at the higher Zn^2+^ concentration range, immediately before the exothermic reaction plateau, a substantial endothermic event was observed to arise. Based on our analysis, we strongly suggest that the first process is a direct cause of Zn^2+^-induced dimerization of ggNucb2, and the second is due to the formation of higher-order oligomers or even precipitation that is typical for this protein under excess Zn^2+^. In contrast, the hsNucb2-Zn^2+^ interaction is characterized by a threefold increased heat rate. The endothermic process caused by Zn^2+^-dependent dimerization was not observed, indicating *a priori* dimerized protein in the apo form. Nonetheless, significant positive heats at the plateau of the titration isotherm were observed, as in the case of ggNucb2. Such endothermic processes might arise from Nucb2 oligomerization. It is commonly observed that in the presence of excess Zn^2+^, a higher assembly of protein oligomers may be formed. Similar thermodynamic properties associated with oligomerization in the presence of Zn^2+^ were observed for insulin monomers [88], soybean ASR protein [78] and one of the late embryogenesis abundant proteins AtLEA4-5 [98]. Notably, the hsNucb2-Zn^2+^ interaction is different from that observed for hsNucb1. Nucb1, the paralogue of Nucb2, was previously characterized [48] as a Zn^2+^ sensor with a high affinity for Zn^2+^ with two Zn^2+^-binding sites (*K*_d_ = 32 nM). In contrast, the fitted complex formation constants for both Zn^2+^-binding sites of hsNucb2 are in the range of 10^-5^ M and seem to be significantly lower than those of Nucb1 [48], and hsNucb2 is characterized as a weak Zn^2+^-binding protein, as Zn^2+^ is tightly buffered in the cell at concentrations in the low nanomolar to picomolar range [99]. Nucb2 amino acid sequences were determined to possess only one putative Zn^2+^ binding site, which might implicate these protein differences [48]. Taken together, all of the results discussed above suggest that the paralogues might fulfil different functions, despite the 62% amino acid sequence identity of hsNucb1 and hsNucb2 [100].

The N-terminal half of Nucb2 contains two out of three polypeptides derived from the precursor protein *in vivo*, i.e., nesfatin-1 and nesfatin-2. There is accumulating evidence regarding the pleiotropic function of nesfatin-1, which is involved in various processes, such as food intake inhibition [38], glucose homeostasis [45], cardiovascular functions [101], and stress behaviour [102]. The role of nesfatin-2 is unknown to date. Notably, a sequence-based analysis of Nucb2s performed with Aggrescan [103] and FoldAmyloid [104], web-based software for the prediction of aggregation-prone segments in protein sequences, identified the aggregation propensity regions within Nucb2s (Fig. S7). These regions were located primarily at the N-terminal half of both homologues. Further research is required to elucidate the full role of the N-terminal half of Nucb2 in this process and its implications. The question remains whether nesfatin-1 and nesfatin-2, either separate from each other or as a part of a full-length precursor protein, might be involved in an aggregation process more efficiently. What is the role of Zn^2+^ binding? Can we link this aggregation propensity of Nucb2 with those exhibited by other amyloidogenic proteins? Notably, Nucb1, a paralogue of Nucb2, has been shown to be a chaperone-like amyloid binding protein [105], [106], [107]. Nucb1 binding stabilizes the protofibril intermediate of such proteins as IAPP, αβ, transthyretin, and α-synuclein [105], [106], [107]. Nucb1 prevents fibril formation at any stage of this process [105], [107]. However, Ca^2+^ binding leads to disruption of the anti-amyloidogenic activity of Nucb1 [105], [107], suggesting that both proteins might play similar roles. Nevertheless, our study indicated substantial differences between the mechanisms of action of these two paralogues.

We also analysed the thermodynamics of Ca^2+^ binding to Nucb2s. ITC revealed that both Nucb2s contain two Ca^2+^-binding sites, which is in keeping with previously obtained HDX-MS results [49]. The Ca^2+^ binding process differs for both Nucb2s. The enthalpy of Ca^2+^ binding is considerably lower for ggNucb2, which might be caused by the varied oligomeric state of apo-Nucb2s. Previous SV-AUC data showed that ggNucb2 undergoes dimerization in the presence of Ca^2+^ [49]. However, hsNucb2 in the apo- and holo-(Ca^2+^) states is dimeric [49].

In conclusion, our results showed that Nucb2s bind Zn^2+^ and Mg^2+^, which affects the structure of Nucb2s. We surmise that the destabilization of the N-terminal half of Nucb2s enables intermolecular interactions, which promote the oligomerization/fibrilization of Nucb2s. Our previous and current research revealed that the structure of Nucb2s is divided into two parts: the Zn^2+^-sensitive N-terminal half (consisting of nesfatin-1 and -2) and the Ca^2+^-sensitive C-terminal half (consisting of nesfatin-3). Structural changes to Nucb2s in the presence of appropriate metal ions probably enable the protein to fulfil different physiological functions. However, further experiments are necessary to elucidate the molecular mechanisms underlying the metal ion-dependent functions of the Nucb2s.

## 4 Materials and methods

### 4.1 Chemicals

All buffers were prepared at room temperature. Buffer A contained 50 mM Na_2_HPO_4_·2 H_2_O and 300 mM NaCl (pH 7.0). Buffer B was 50 mM Na_2_HPO_4_·2 H_2_O, 300 mM NaCl, and 200 mM imidazole (pH 7.0). Buffer C contained 20 mM Tris-HCl, 150 mM NaCl (pH 7.5). Buffer D contained 20 mM HEPES, 150 mM NaCl (pH 7.5). The quench buffer contained 2 M glycine (pH 2.53). DNase I and RNase A, ZnCl_2_·2 H_2_O, MgCl_2_·6 H_2_O, CaCl_2_·2H_2_O and EDTA were purchased from Sigma-Aldrich. All other chemicals were of analytical grade and commercially available.

### 4.2 Protein expression and purification

The ggNucb2 and hsNucb2 coding sequences were amplified by polymerase chain reaction (PCR) with One-Fusion DNA polymerase (Gene ON) and with primers introducing the SacI and HindIII restriction sites. The purified PCR products were digested with SacI and HindIII restriction enzymes and ligated into the pQE-80L/HRV3C vector, modified in our laboratory to introduce the HRV 3C protease cleavage sequence at the N-terminus of the target protein. This approach enabled further investigation of a tag-free protein with only four extra amino acid residues (Gly-Pro-Glu-Lys) added eventually at the N-terminus, which are derived from the HRV 3C protease cleavage sequence. The sequences in the DNA constructs were verified by Sanger sequencing (Genomed S.A.). Both Nucb2s were expressed and purified as reported previously [49]. Before the final size exclusion chromatography (SEC), proteins were digested with HRV 3C protease using a 100:1 (w/w) protein-to-protease ratio overnight at 4 °C and concentrated on Amicon Ultra-4 centrifugal filter units (Millipore). Proteins purified for ITC analyses were incubated with 5 mM EDTA for 1 h before SEC and eluted from the column with buffer D. The elution fractions were monitored by A_280_ measurements. The protein concentrations were measured at 280 nm utilizing the following extinction coefficients: ggNucb2 – 21,430 M^−1^ × cm^−1^ and hsNucb2 – 24,410 M^−1^ × cm^−1^, which were estimated from the amino acid sequence. The homogeneity and chemical identity of targeted proteins were evaluated by mass spectrometry and SDS-PAGE analyses.

### 4.3 Circular dichroism (CD)

Far-UV CD spectra were obtained using a JASCO J-815CD spectropolarimeter equipped with a Peltier-type temperature controller (Type Control System, JASCO) with a 1-mm path length quartz cuvette. The measurements were performed for both Nucb2s in buffer C in the absence (5 mM EDTA) and presence of 10 mM MgCl_2_ or 100 µM, 200 µM and 300 µM ZnCl_2_. Data were recorded at 20 °C from 300 nm to 195 nm with a scanning speed of 50 nm/min. Three scans were performed with a step size of 0.5 nm and a bandwidth of 1 nm and averaged for each condition. The solvent background was subtracted from each spectrum for all the measurements. The secondary structure composition was calculated using the CDNN package [56].

### 4.4 Fluorescence spectroscopy

The measurements were carried out at 20 °C utilizing a Fluorolog-3 spectrofluorometer (Horiba Jobin Yvon Inc.). Fluorescence spectra of Trp residues were recorded for 2 µM Nucb2s in buffer C either in the presence of 5 mM EDTA or 10 mM MgCl_2_ and 100 µM, 200 µM and 300 µM ZnCl_2_. Both homologues were incubated in the appropriate solution for 30 min before the measurement. The spectra were recorded between 310 and 500 nm, with an excitation wavelength of 295 nm. The obtained spectra were corrected for buffer contribution.

### 4.5 Sedimentation velocity analytical centrifugation (SV-AUC)

Nucb2 samples were subjected to AUC in the absence and presence of different metal cations. Sedimentation velocity experiments were performed at 20 °C and 50,000 rpm on a Beckman Coulter Proteome Lab XL-I ultracentrifuge (Beckman Coulter Inc.) equipped with an An-60Ti rotor. Protein samples (0.50 mg/ml, 0.75 mg/ml and 1.0 mg/ml) were prepared in buffer C supplemented with either 5 mM EDTA, 10 mM MgCl_2_ or 100 µM, 200 µM or 300 µM ZnCl_2_. The wavelength used for sample detection was 280 nm. The protein partial specific volumes (0.727 ml/g and 0.732 ml/g for ggNucb2 and hsNucb2, respectively), buffer density (1.0059 g/ml, 1.0057 g/ml and 1.0060 g/ml for EDTA-, MgCl_2_- or ZnCl_2_-containing buffer, respectively) and viscosity (1.0265 mPa × s, 1.0258 mPa × s and 1.0228 mPa × s for EDTA-, MgCl_2_- or ZnCl_2_-containing buffer, respectively) were calculated with SEDNTERP [108]. Time-corrected data were analysed with Sedfit software (version 16.1) using the built-in continuous sedimentation coefficient distribution model c(s). Maximum-entropy regularization of the c(s) model was set to a confidence level of 0.68 [109], [110].

### 4.6 Hydrogen-deuterium exchange coupled with mass spectrometry (HDX-MS)

The HDX-MS experiments were performed as described previously [49], but to obtain the highest sequence coverage, the Nepenthesin-2 enzyme was used instead of pepsin for online protein digestion, utilizing a 2.1 mm × 20 mm Nepenthesin-2 column (AffiPro). HDX-MS was performed for both Nucb2s in the presence of 5 mM EDTA, 10 mM MgCl_2_ and 100 µM ZnCl_2_. Fifteen microlitres of ggNucb2 or hsNucb2 (95 µM of each) was diluted with 35 µl of buffer C (H_2_O-based) in the absence and presence of the appropriate additive. The proteins were acidified by mixing with 10 µl of H_2_O-based quench buffer containing 2 M glycine, pH 2.5. Proteins were digested online with a 2.1 mm × 20 mm Nepenthesin-2 column (AffiPro) at 20 °C. The generated peptides were trapped on a VanGuard precolumn (C18, 2.1 mm × 5 mm; Waters) and desalted for 1.5 min at a flow rate of 120 µl/min of solvent A (0.07% formic acid in Milli-Q water) controlled by the nanoACQUITY Auxilary Solvent Manager. Subsequently, peptides were resolved by a reverse-phase column ACQUITY UPLC BEH C18 column (2.1 mm × 50 mm, Waters) using a 6–40% gradient of acetonitrile in 0.01% formic acid at a flow rate of 90 µl/min, controlled by the nanoACQUITY Binary Solvent Manager.

### 4.7 Isothermal titration calorimetry (ITC)

The binding of Ca^2+^, Zn^2+^ and Mg^2+^ to Nucb2s was monitored using a Nano-ITC calorimeter (TA Waters, USA) at 25 °C with a cell volume of 1 ml. All experiments were performed in buffer D. The Nucb2 (titrate) concentration was 0.032–0.075 mM, whereas the metal ion (titrant) concentration was 1–6 mM. Concentrations of titrate and titrant were adjusted to obtain the best isotherms for proper analysis of equilibria and to highlight different processes throughout the titration experiment. After temperature equilibration, successive injections of the titrant were made into the reaction cell in 5.22-μl increments at 300-s intervals with stirring at 250 rpm. Control experiments to determine the heats of titrant dilution were performed using identical injections of Ca^2+^/Zn^2+^/Mg^2+^ in the absence of protein. The net reaction heat was obtained by subtracting the dilution heat from the corresponding total heat of reaction.

The titration data were analysed using NanoAnalyze (version 3.3.0), NITPIC (version 1.2.7) [111], [112] and SEDPHAT (version 15.2b) [113]. First, data were preprocessed using NanoAnalyze software dedicated to the Nano-ITC calorimeter. Second, data integration and baseline subtraction were conducted using NITPIC freeware. Afterwards, integrated data were fitted with SEDPHAT. Depending on the analysed variant, different models of interaction were used for fitting; however, all of the variants titrated with Ca^2+^, as well as hsNucb2 titrated with Zn^2+^, gave good fits for the standard ABB model, where a unit of protein possesses two symmetrical binding sites: *A + B + B* ⇌ *AB + B* ⇌ *ABB* [113]. Another model that was used was one that takes into account the self-association of monomers into dimers that coincides with metal ion binding: *(A + A) + B + B* ⇌ *AB + A + B* ⇌ *(AA)B + B* ⇌ *(AA)BB*. The model was used to simulate the first tendency to gradually increase the exothermic process that was visible mostly for ggNucb2 titrated with Ca^2+^ but was very discrete for hsNucb2 samples. Last, Nucb2s titrated with Mg^2+^ were best fitted with a simple *A + B* ⇌ *AB* model, which suggests that hsNucb2 and ggNucb2 bind only one Mg^2+^ ion. Data were fitted first with constrained values obtained from prefitting in NanoAnalyze and subsequently revised in SEDPHAT with floating parameters of an incompetent fraction of protein and log*K*_x_ with the corresponding Δ*H*_x_ under the appropriate model of interaction. The error estimates for the fitting results were produced using Monte Carlo analysis, individually for each experiment, with 500 iterations and a 0.9 level of confidence. For hsNucb2 samples titrated with Ca^2+^ and Zn^2+,^ single isotherms fitted to the ABB model gave good and reproducible results, but ggNucb2 samples titrated with Ca^2+^ gave significantly more complex results and were analysed using three different approaches: i) “narrow range” fit that focuses on the second isotherm alone and ii) “broad range” fit that fits both processes observable throughout the titration.

### 4.8 Small-angle X-ray scattering (SAXS)

SAXS profiles for both Nucb2s in solution were obtained by the use of XEUSS 2.0 SAXS/WAXS system (Xenocs, Grenoble, France). The ggNucb2 and hsNucb2 samples (protein concentration of 3.5 mg/ml) with 5 mM EDTA or 10 mM Ca^2+^, 100 µM Zn^2+^ and 10 mM Mg^2+^ and reference sample (BSA, bovine serum albumin) were injected into the flow cell and exposed to X-ray radiation (gallium Kα emission of 9.2 keV) produced by a MetalJet D2 microfocus generator (Excillum AB, Kista, Sweden). For each sample, 10 independent frames (exposure time 600 s per frame) were recorded using a PILATUS3 1 M detector (DECTRIS Ltd., Baden-Daettwil, Switzerland). Scattering data for ggNucb2 and hsNucb2 were integrated using Foxtrot [114] and processed using the SAS data analysis module from the ATSAS package [115].

### 4.9 Transmission electron microscopy (TEM)

Microscopic observations were performed using a Hitachi H-800 conventional transmission electron microscope (Hitachi Hightech, Japan). An accelerating voltage of 150 kV and the EMSIS Quemesa CCD camera were used. The samples were prepared similar to protocols [116], [117]. Commercial carbon on a copper mesh 200 grid (AGS160) was used (Agar Scientific, United Kingdom). Before each experiment, Nucb2s (150 µM) in buffer C were incubated with 300 µM Zn^2+^, 10 mM Ca^2+^ and 10 mM Mg^2+^ at 4 °C for two weeks. Subsequently, 20 µl of the Nucb2s were applied on a strip of parafilm or a silicone pad, and 150 µl of deionized water was added. The grid was placed on a drop of sample for adhesion, and after 30 s, it was blotted on filter paper. The grid was immediately transferred for water droplets for 60 s on each of them. After removing the last portion of water, the sample was blotted with filter paper and immediately covered with 4 µl of 2% uranyl acetate. After 30 s of staining, the grid was blotted and allowed to dry in an ajar petri dish for at least 24 h. The same protocol was used to determine the morphology of the supernatant and precipitate of ggNucb2 upon the addition of 300 µM Zn^2+^ after the AUC experiment.

At the preparation stage, we compared usage of the carbon films straight out of the box and glow-discharged, different protein concentrations, sample washing with buffer and water as well as staining with uranyl acetate and uranyl formate. We are aware that the use of water as a medium for the long-term removal of excess concentrated proteins may cause changes in its structure, just as the use of non-glow-discharged grids may cause excessive protein complexation. For this reason, we note that the procedure used is only comparative and shows the tendency to produce protein complexes in the specific buffers.

## Funding

This work was supported by the National Science Centre Grant 2018/29/B/NZ1/02574 (A.O.). The HDX-MS equipment used was sponsored by the Centre for Preclinical Research and Technology (CePT, a project cosponsored by the European Regional Development Fund and Innovative Economy), the National Cohesion Strategy of Poland (POIG.02.02.00-14-024/08-00), and the National Multidisciplinary Laboratory of Functional Nanomaterials (POIGT.02.02.00-00-025/09-00).

## CRediT authorship contribution statement

**Dominika Bystranowska:** Investigation, Methodology, Project administration, Writing - original draft, Writing - review & editing. **Anna Skorupska:** Investigation, Methodology, Writing - original draft, Writing - review & editing. **Katarzyna Sołtys:** Investigation, Methodology, Writing - review & editing. **Michał Padjasek:** Investigation, Methodology, Writing - original draft, Writing - review & editing. **Artur Krężel:** Methodology, Writing - review & editing. **Andrzej Żak:** Investigation, Methodology, Writing - original draft, Writing - review & editing. **Magdalena Kaus-Drobek:** Investigation, Methodology, Writing - review & editing. **Michał Taube:** Investigation, Methodology, Writing - review & editing. **Maciej Kozak:** Methodology, Writing - review & editing. **Andrzej Ożyhar:** Methodology, Funding acquisition, Project administration, Supervision, Writing - original draft, Writing - review & editing.

## Declaration of Competing Interest

The authors declare that they have no known competing financial interests or personal relationships that could have appeared to influence the work reported in this paper.
