# Supplementary material for: Nucleobindin-2 consists of two structural components: The Zn2+-sensitive N-terminal half, consisting of nesfatin-1 and -2, and the Ca2+-sensitive C-terminal half, consisting of nesfatin-3
Source: Comput Struct Biotechnol J. 2021 Jul 30;19:4300–18. doi: 10.1016/j.csbj.2021.07.036 (PMC8361300; doi:10.1016/j.csbj.2021.07.036)
Supplement: Supplementary data 1 [file mmc1.docx]

**Supplemental Figures**

**Fig. S1**

**
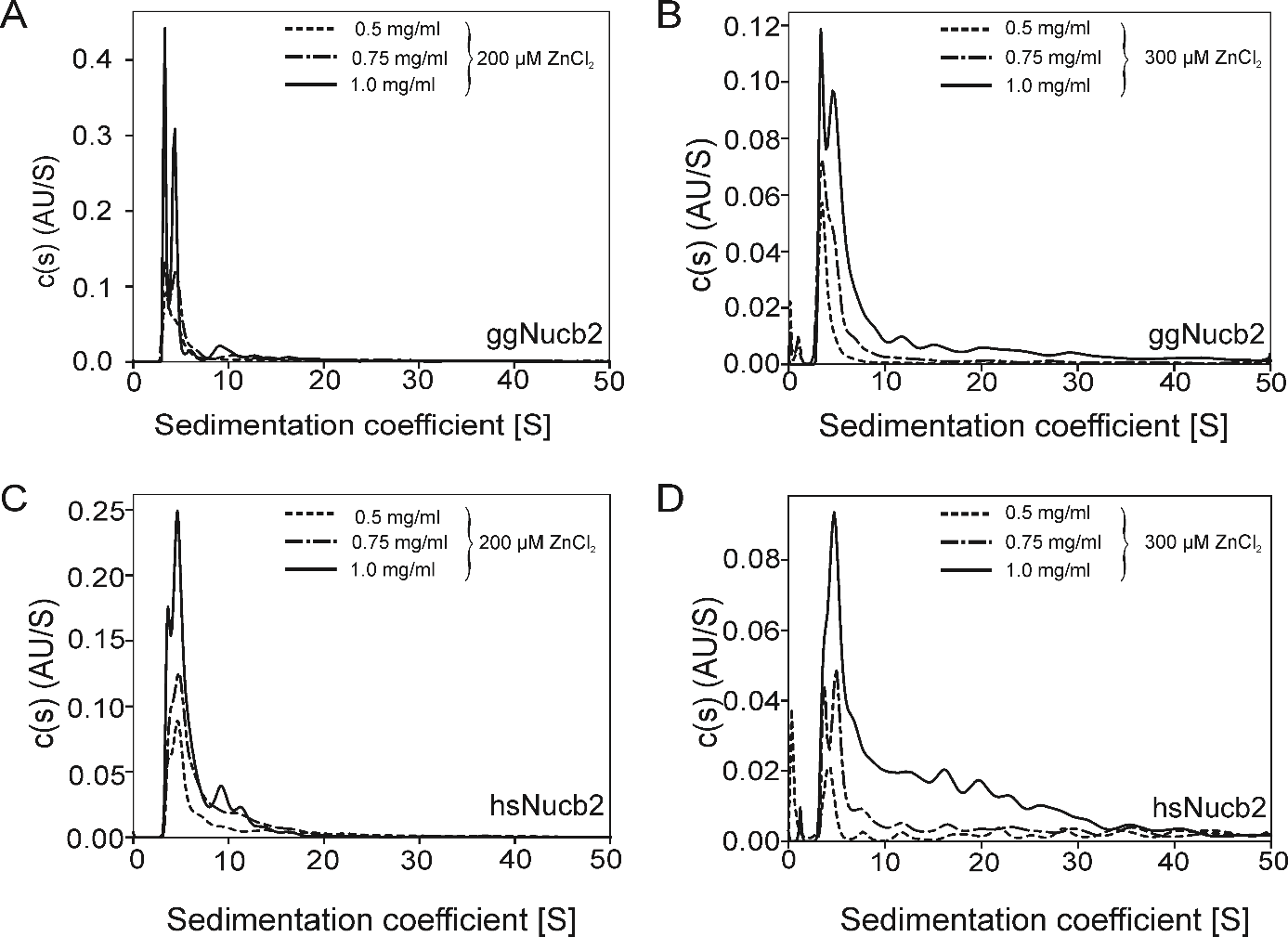
**

**Fig. S1.** Size distribution characterization of Nucb2s in the presence of varied Zn^2+^ concentrations. **(A)** The sedimentation coefficient c(s) distribution for ggNucb2 in the presence 200 µM ZnCl_2_, **(B)** The sedimentation coefficient c(s) distribution for ggNucb2 in the presence of 300 µM ZnCl_2_, **(C)** The sedimentation coefficient c(s) distribution for hsNucb2 in the presence of 200 µM ZnCl_2_, **(D)** The sedimentation coefficient c(s) distribution for hsNucb2 in the presence of 300 µM ZnCl_2_.

**Fig. S2.**

**
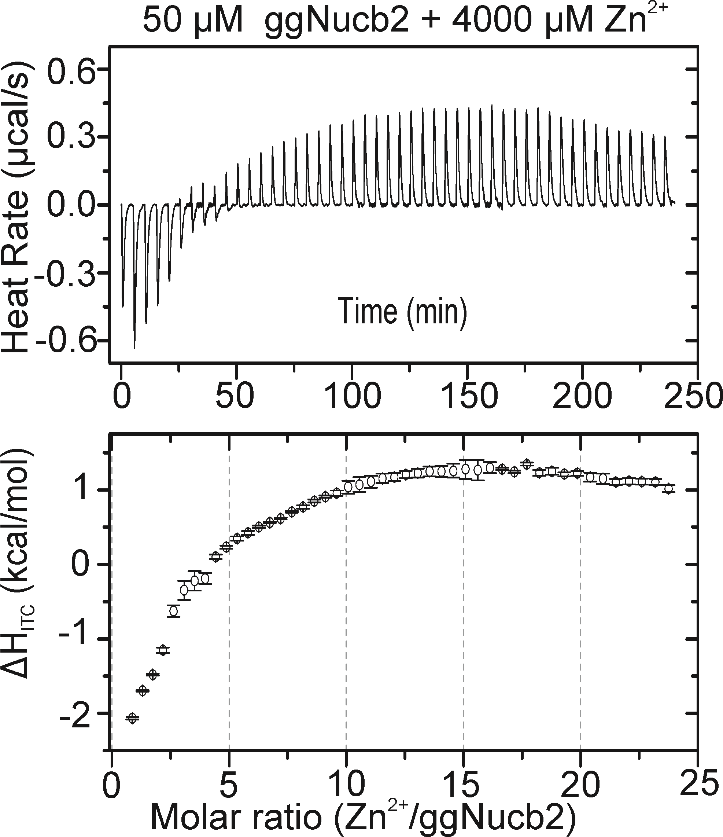
**

**Fig. S2.** ITC profiles for Zn^2+^ binding to ggNucb2. The top panel shows the baseline-subtracted thermograms. The bottom panel represents the binding isotherm. The errors on the Y axis correspond to standard deviations from the baseline subtraction procedure. All the measurements were obtained in 20 mM HEPES and 150 mM NaCl (pH 7.5).

**Fig. S3.**

**
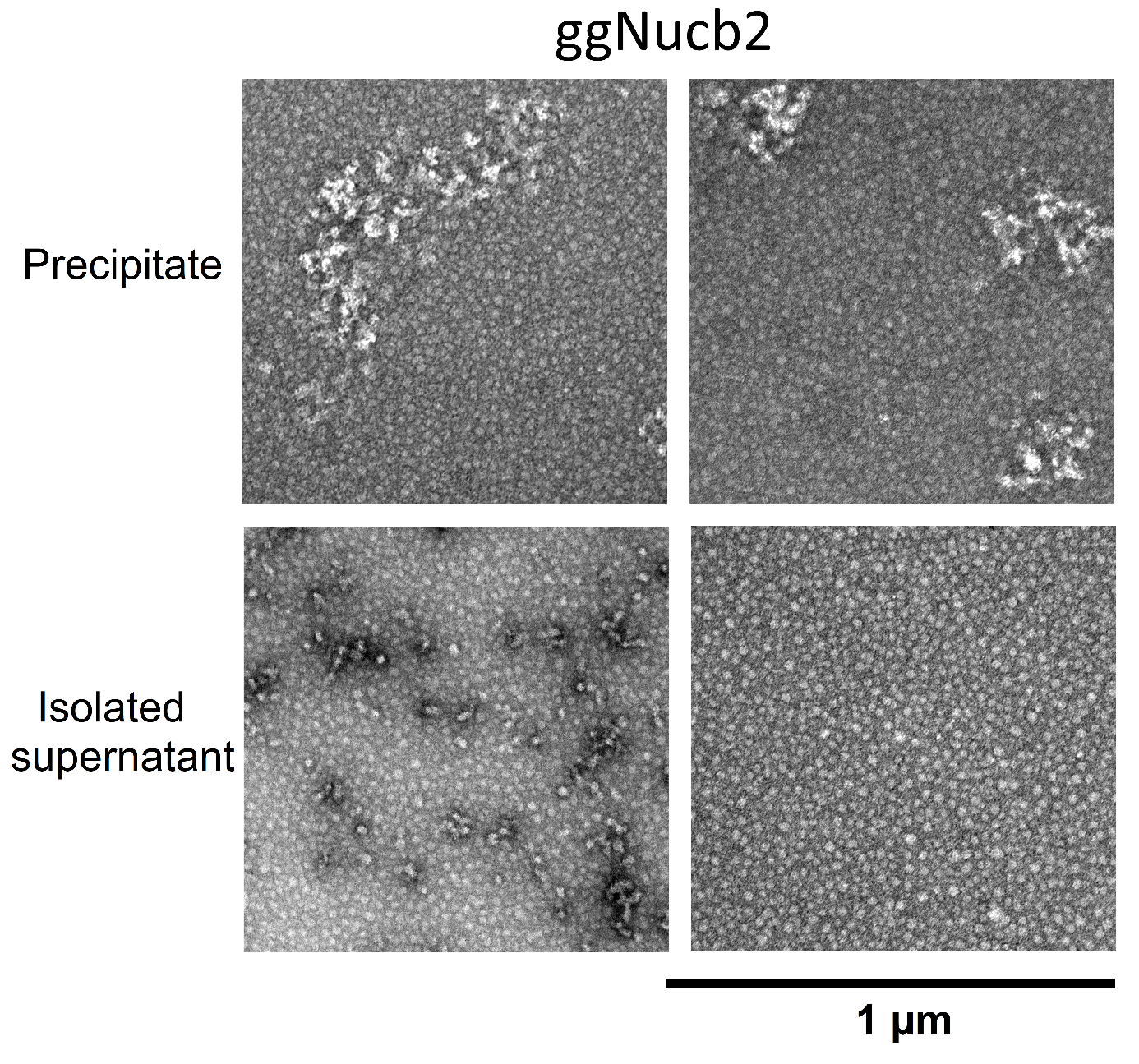
**

**Fig. S3**. The morphology of the Nucb2 oligomers formed in the presence of 300 µM Zn^2+^. Representative TEM images of ggNucb2 precipitate and isolated supernatant. The scale bars represent 1 µm.

**Fig. S4.**

**
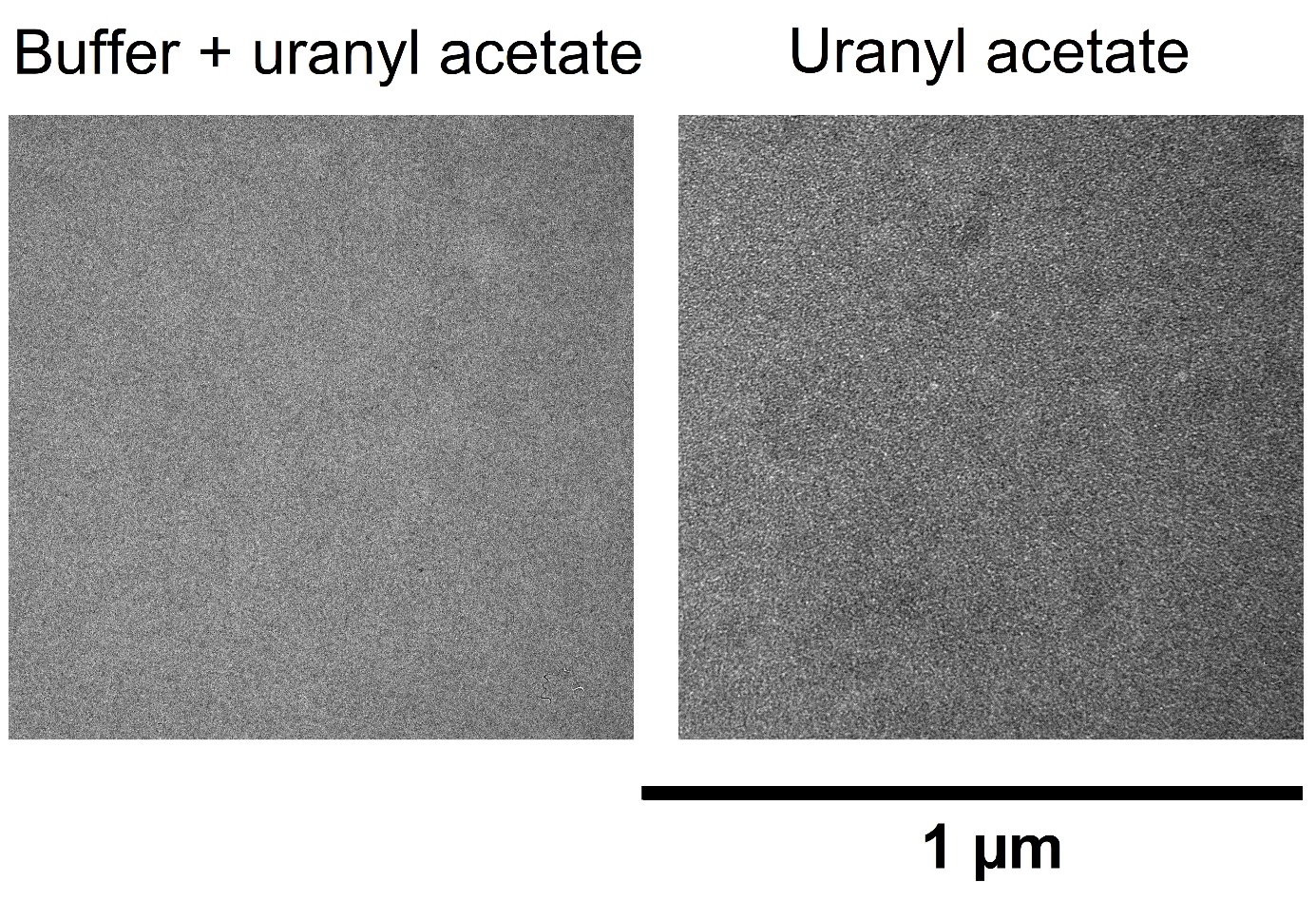
**

**Fig. S4**. The control TEM images. The purity of the buffer and staining agent was checked. The scale bar indicates 1 µm.

**Supplemental tables**

**Table S1.** Thermodynamic parameters of Ca^2+^ complexation of ggNucb2 derived from fitting data to the AA BB model of interactions accounting for ligand-mediated dimerization.

| Parameter | ggNucb2 + Ca^2+^  (broad range) |
| --- | --- |
| [Nucb2] (µM) | 50 |
| [Ca^2+^] (µM) | 4000 |
| Χ^2^ | 1.2 |
| *K*_d(aa)_ (µM) | 0.016 ±0.027 |
| ΔH_aa_ (kcal/mol) | 7.21 ±0.11 |
| TΔS_aa_ (kcal/mol) | 17.88 |
| *K*_d(ab)_ (µM) | 60.14 ±0.36 |
| ΔH_ab_ (kcal/mol) | -2.82 ±0.29 |
| TΔS_ab_ (kcal/mol) | 2.94 |
| *K*_d((aa)b)_ (µM) | 13.11 ±0.78 |
| ΔH_(aa)b_ (kcal/mol) | 0.96 ±0.19 |
| TΔS_(aa)b_ (kcal/mol) | 7.62 |
| *K*_d(b(aab))_ (µM) | 10.13 ±0.81 |
| ΔH_baab_ (kcal/mol) | -22.89 ±1.01 |
| TΔS_baab_ (kcal/mol) | -16.08 |
| coop AAB/AB | 1.7 |
| coop BAAB/AAB | 0.2 |

Nucb2 – nucleobindin 2; Χ^2^ – chi-square; X_aa_ (*K*_daa_, ΔH_aa_, TΔS_aa_) – thermodynamic parameters of dimer formation; X_ab_ – thermodynamic parameters of the first binding event; X_(aa)b_ – thermodynamic parameters of metal binding by the Nucb2 dimer; X_b(aab)_ - thermodynamic parameters of the second metal binding by the Nucb2 dimer; coop AAB/AB – cooperativity index for ligand-induced dimerization; coop BAAB/AAB – cooperativity index for enhancement of the second binding by the first one.

**Zn^2+^-dependent local stability of Nucb2s shown by limited proteolysis**

**Results**

CD spectroscopy indicated that Zn^2+^ has an impact on the secondary structure of Nucb2s, which results in the folding of Nucb2s. To further assess the effect of Zn^2+^ binding on the structure of Nucb2s, we utilized the limited proteolysis technique. The digestion of Nucb2s in the absence and presence of Zn^2+^ was conducted by serine protease glutamyl endopeptidase Glu-C (V8) from *Staphylococcus aureus.* V8 displays a specificity for hydrolysis at the C-terminus of Glu or Asp residues [1]. Limited proteolysis occurs mostly at flexible loops rather than rigid secondary structures such as α-helices [2–4]. Therefore, limited proteolysis is frequently used to probe conformational changes. The proteolysis reactions in the absence and presence of Zn^2+^ are shown in Fig. S5. The hydrolysis was quenched at different time points from 0 to 240 minutes, and the reaction products were analysed by SDS-PAGE. Digestion of ggNucb2 in the absence of ZnCl_2_ (Fig. S5A) resulted in the accumulation of several fragments, migrating at 45 kDa and 35 kDa. During prolonged reaction, the 45 kDa fragments are probably digested further, leading to increased accumulation of 35 kDa fragments. Interestingly, incubation of ggNucb2 with 100 µM ZnCl_2_ resulted only in the accumulation of fragments migrating at 45 kDa, which suggests that ggNucb2 exhibits more resistance to protease V8 digestion in the presence of Zn^2+^. Digestion of hsNucb2 (Fig. S5C and S5D) occurred in a slightly different manner. hsNucb2 in the absence of ZnCl_2_ was cleaved into fragments migrating at 35 kDa and some small molecular weight fragments. In contrast, in the presence of Zn^2+^, hsNucb2 acquired resistance to protease digestion. The bands migrating at 35 kDa appear at longer incubation times (after 60 minutes) on a smaller scale, and the band of untreated hsNucb2 remains intact. Furthermore, we facilitated the digestion of Nucb2s in the presence of various concentrations of ZnCl_2_ (0 to 100 µM) (Fig. S6A and S6B). The proteolysis reactions indicate that both Nucb2s in the presence of lower concentrations of ZnCl_2_ (0 to 10 µM) are susceptible to proteolysis. However, in the presence of 100 µM ZnCl_2_, protection against V8 digestion was acquired. These results showed that the conformational flexibility of both Nucb2s is high and that in the presence of Zn^2+,^ both homologues undergo conformational changes, leading to a more compact/rigid form.

**
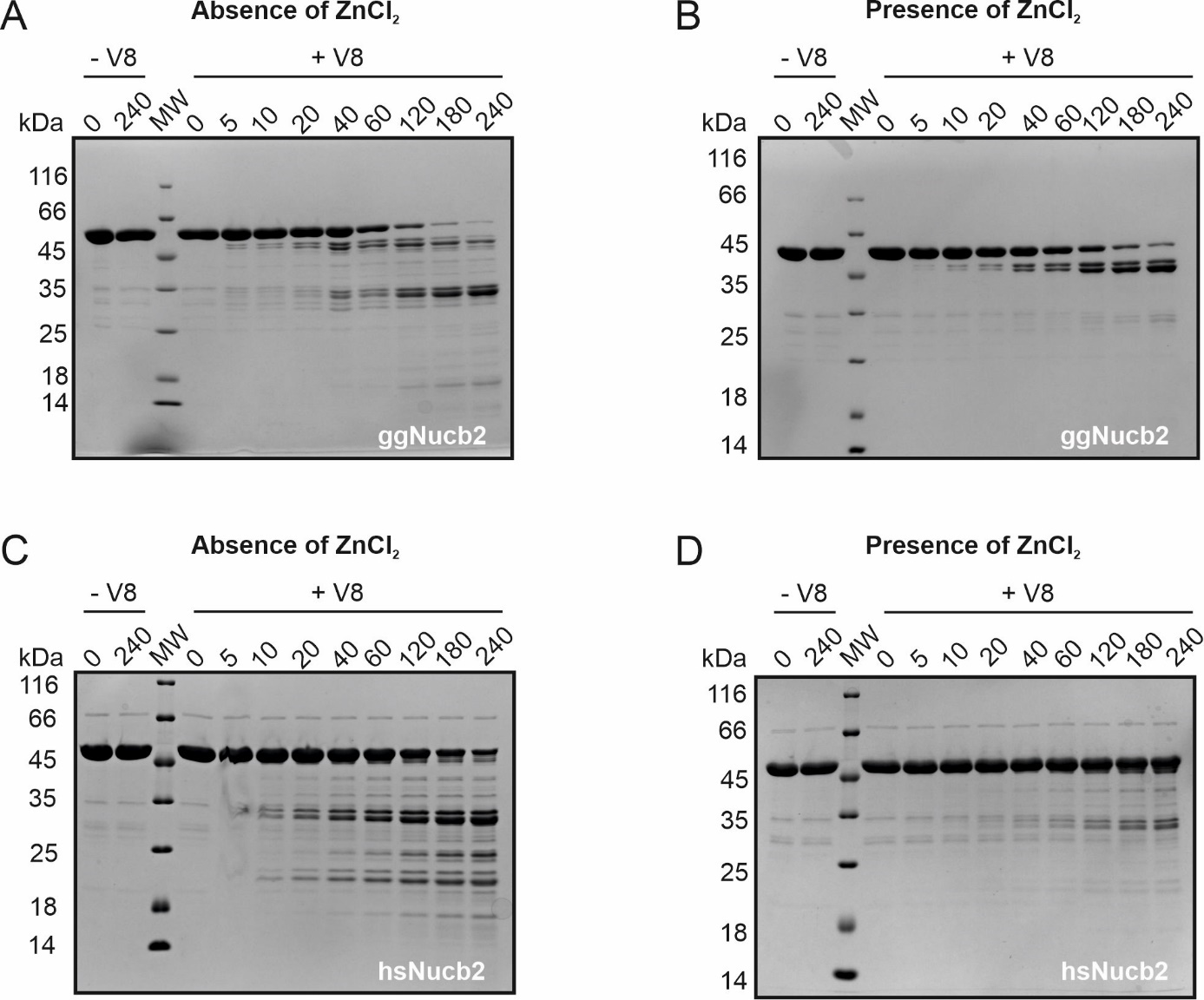
**

**Fig. S5. SDS-PAGE analysis of V8 digestion of Nucb2s. (A)** ggNucb2 in the presence of EDTA, **(B)** ggNucb2 in the presence of Zn^2+^, (**C)** hsNucb2 in the presence of EDTA, **(D)** hsNucb2 in the presence of Zn^2+^. The proteolysis reactions occurred at 20 °C at an enzyme-to-substrate ratio of 1:5,000 over 240 minutes. MW – molecular mass marker; -V8 – control reactions with no protease.


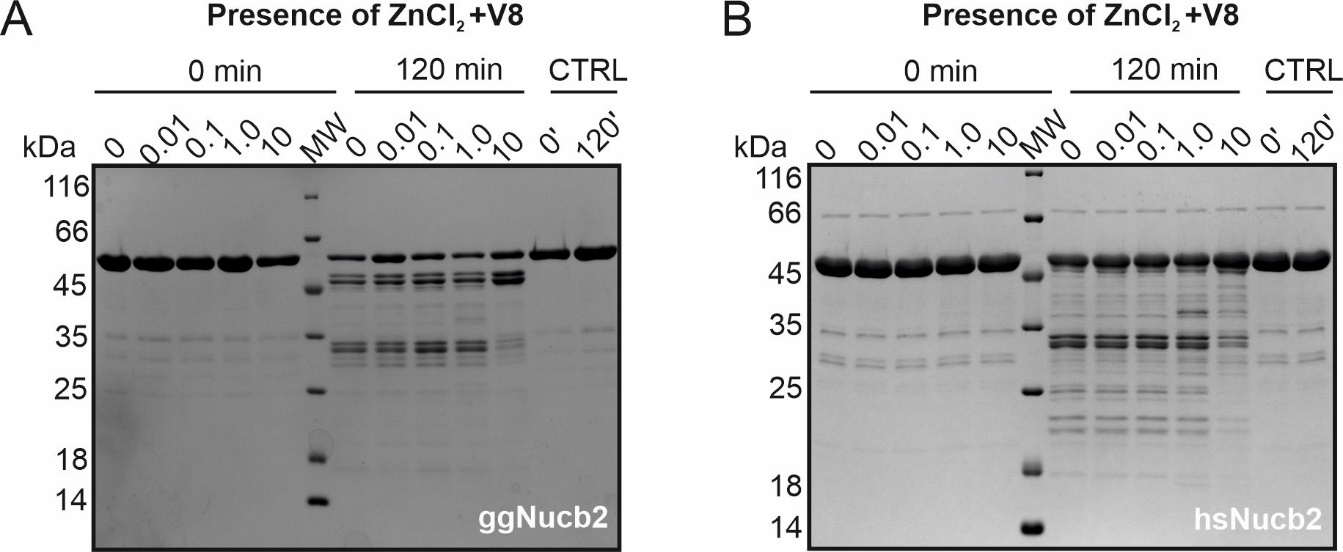


**Fig. S6. SDS-PAGE analysis of V8 digestion in the presence of varied concentrations of Zn^2+^ (0–100 µM). (A)** ggNucb2, **(B)** hsNucb2. Proteolysis was carried out at 20 °C at an enzyme-to-substrate ratio of 1:5,000 for 120 minutes. MW – molecular mass marker; CTRL – control reactions with no protease.

**Methods**

ggNucb2 and hsNucb2 (10 µM) were digested with endopeptidase Glu-C (V8) (1:5000 w/w) in buffer C in the presence of 5 mM EDTA and varying amounts ZnCl_2_ (0 to 100 µM) at 20 °C. Aliquots of 10 µl were removed at various time points, and the reaction was stopped by the addition of SDS loading buffer. The samples (3.75 µg) were separated by SDS-PAGE and stained with Coomassie Blue.

**Aggregation propensity analysis**

The amyloidogenic propensity prediction of Nucb2s was performed utilizing the Aggrescan [5] and FoldAmyloid algorithms [6].

**
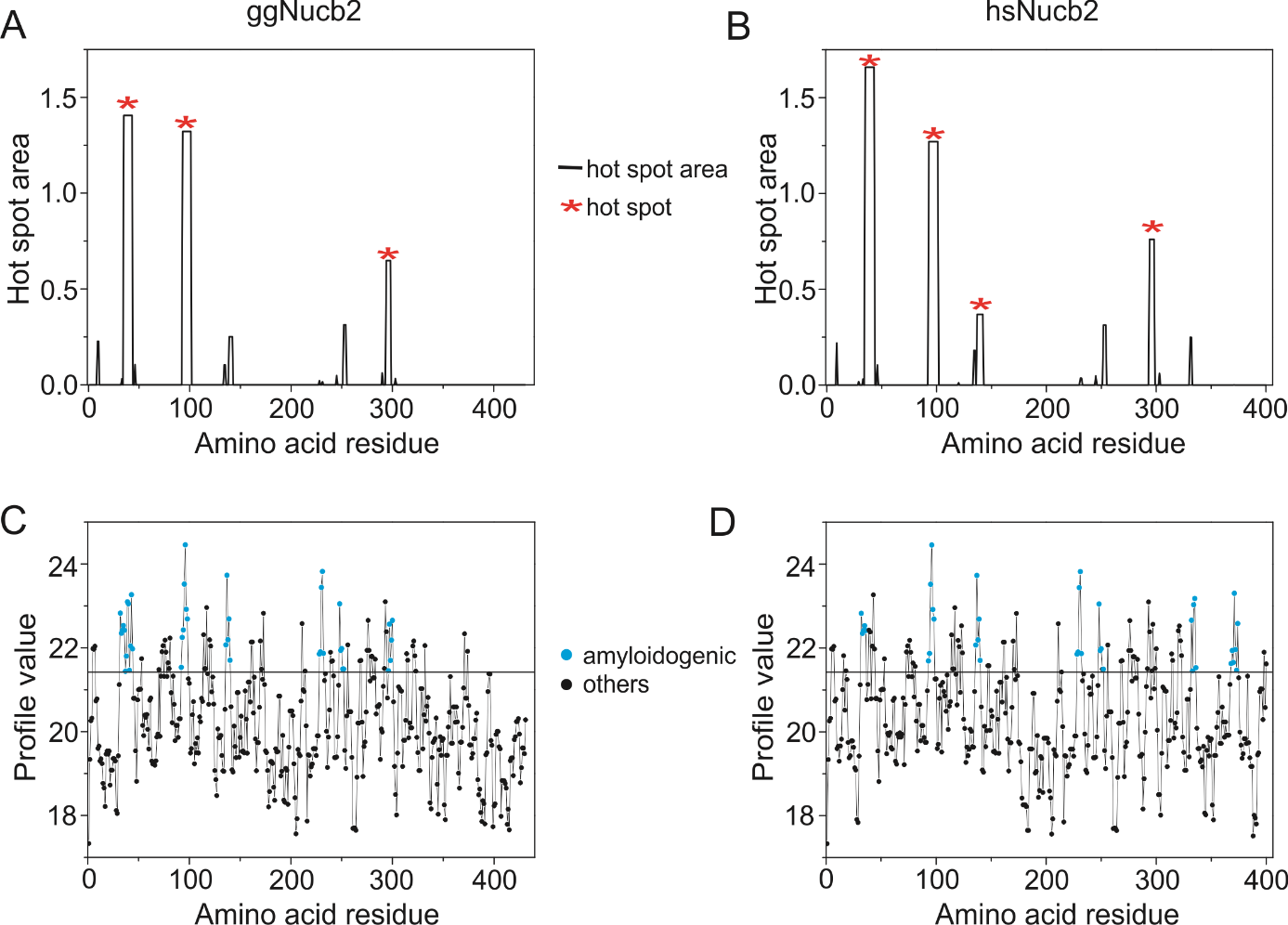
**

**Fig. S7. Aggregation predisposition analysis of Nucb2s.** The aggregation propensity predicted by Aggrescan for ggNucb2 **(A)** and hsNucb2 **(B)**. The aggregation hot spots predicted by Aggrescan are shown by red asterisks, and the hot spot areas are shown by solid black lines. [5]. The hot spots are determined as the sequence of 5 or more continuous amino acid residues with scores larger than a threshold and without a proline residue (aggregation breaker) [5,7]. The amyloidogenic regions in the ggNucb2 **(C)** and hsNucb2 **(D)** locations predicted by FoldAmyloid [6]. The blue dots illustrate aggregation-prone amino acid residues. The regions of proteins are predicted to be amyloidogenic if the residues of these regions have a greater predicted value than the threshold (21.4) and the regions are greater than or equal to the size of the frame used [6]. The threshold value (21.4) was determined on the basis of a merged database of amyloidogenic proteins [6].

**Literature**

1. Drapeau GR, Boily Y, Houmard J. Purification and properties of an extracellular protease of Staphylococcus aureus. J Biol Chem. 1972;247: 6720–6726.

2. Fontana A, Fassina G, Vita C, Dalzoppo D, Zamai M, Zambonin M. Correlation between sites of limited proteolysis and segmental mobility in thermolysin. Biochemistry. 1986;25: 1847–1851.

3. Receveur-Bréchot V, Bourhis J-M, Uversky VN, Canard B, Longhi S. Assessing protein disorder and induced folding. Proteins Struct Funct Bioinforma. 2005;62: 24–45. doi:10.1002/prot.20750

4. Neurath H. Proteolytic processing and physiological regulation. Trends Biochem Sci. 1989. doi:10.1016/0968-0004(89)90061-3

5. Conchillo-Solé O, de Groot NS, Avilés FX, Vendrell J, Daura X, Ventura S. AGGRESCAN: A server for the prediction and evaluation of “hot spots” of aggregation in polypeptides. BMC Bioinformatics. 2007;8: 1–17. doi:10.1186/1471-2105-8-65

6. Garbuzynskiy SO, Lobanov MY, Galzitskaya O V. FoldAmyloid: A method of prediction of amyloidogenic regions from protein sequence. Bioinformatics. 2009. doi:10.1093/bioinformatics/btp691

7. Williams AD, Portelius E, Kheterpal I, Guo JT, Cook KD, Xu Y, et al. Mapping Aβ amyloid fibril secondary structure using scanning proline mutagenesis. J Mol Biol. 2004;335: 833–842. doi:10.1016/j.jmb.2003.11.008
